# Oxysterols protect bovine endometrial cells against pore‐forming toxins from pathogenic bacteria

**DOI:** 10.1096/fj.202100036R

**Published:** 2021-09-27

**Authors:** Thomas J. R. Ormsby, Sian E. Owens, Anthony D. Horlock, Daphne Davies, William J. Griffiths, Yuqin Wang, James G. Cronin, John J. Bromfield, Iain M. Sheldon

**Affiliations:** ^1^ Swansea University Medical School Swansea University Swansea UK; ^2^ Department of Animal Sciences University of Florida Gainesville Florida USA

**Keywords:** cattle, cholesterol, cytoprotection, liver X receptor, uterus

## Abstract

Many species of pathogenic bacteria secrete toxins that form pores in mammalian cell membranes. These membrane pores enable the delivery of virulence factors into cells, result in the leakage of molecules that bacteria can use as nutrients, and facilitate pathogen invasion. Inflammatory responses to bacteria are regulated by the side‐chain‐hydroxycholesterols 27**‐**hydroxycholesterol and 25**‐**hydroxycholesterol, but their effect on the intrinsic protection of cells against pore‐forming toxins is unclear. Here, we tested the hypothesis that 27**‐**hydroxycholesterol and 25**‐**hydroxycholesterol help protect cells against pore‐forming toxins. We treated bovine endometrial epithelial and stromal cells with 27**‐**hydroxycholesterol or 25**‐**hydroxycholesterol, and then challenged the cells with pyolysin, which is a cholesterol‐dependent cytolysin from *Trueperella pyogenes* that targets these endometrial cells. We found that treatment with 27‐hydroxycholesterol or 25**‐**hydroxycholesterol protected both epithelial and stomal cells against pore formation and the damage caused by pyolysin. The oxysterols limited pyolysin‐induced leakage of potassium and lactate dehydrogenase from cells, and reduced cytoskeletal changes and cytolysis. This oxysterol cytoprotection against pyolysin was partially dependent on reducing cytolysin‐accessible cholesterol in the cell membrane and on activating liver X receptors. Treatment with 27**‐**hydroxycholesterol also protected the endometrial cells against *Staphylococcus aureus* α‐hemolysin. Using mass spectrometry, we found 27**‐**hydroxycholesterol and 25**‐**hydroxycholesterol in uterine and follicular fluid. Furthermore, epithelial cells released additional 25**‐**hydroxycholesterol in response to pyolysin. In conclusion, both 27**‐**hydroxycholesterol and 25**‐**hydroxycholesterol increased the intrinsic protection of bovine endometrial cells against pore‐forming toxins. Our findings imply that side‐chain‐hydroxycholesterols may help defend the endometrium against pathogenic bacteria.

**Abbreviations**
25HC: 25‐hydroxycholesterol
27HC: 27‐hydroxycholesterol
ABCA1: ATP binding cassette subfamily A member 1
ACAT: acetyl‐CoA acetyltransferase
LDH: lactate dehydrogenase
LPS: lipopolysaccharide
LXR: liver X receptor

## 1 INTRODUCTION

Parturition is accompanied by bacterial infection of the uterus, and disruption of the protective epithelium of the endometrium, which exposes the underlying stroma. This is a particular problem in cattle, where bacterial infections cause postpartum uterine disease in up to 40% of animals, with a resulting loss of fertility.^1^, ^2^ Many species of bacteria secrete toxins that form pores in cell membranes, leading to cell damage. Preventing disease depends on limiting the damage caused by these pathogens, and on the immune system killing and removing the pathogens.^1^, ^3^ The immune system is regulated, at least in part, by oxysterols.^4^, ^5^, ^6^ However, it is unclear whether oxysterols can also affect the protection of tissue cells against damage by pathogens.

Pore‐forming toxins enable the delivery of other bacterial virulence factors into cells, result in the leakage of molecules that bacteria can use as nutrients, and facilitate bacterial invasion.^7^ Among the many species of bacteria that infect the uterus of cattle post partum, pathogens that secrete pore‐forming toxins include *Trueperella pyogenes* and *Staphylococcus aureus*.^8^, ^9^, ^10^ *T. pyogenes* secretes pyolysin, which is a member of the most common family of pore‐forming toxins, cholesterol‐dependent cytolysins.^7^, ^9^, ^11^ Cholesterol‐dependent cytolysins bind cholesterol in cell membranes, which is accessible when membrane lipids comprise >35 mol% cholesterol.^12^, ^13^, ^14^ Pyolysin is secreted as a 55 kDa monomer and 30 to 50 molecules oligomerize to form an 18 nm internal diameter β‐barrel transmembrane pore.^11^, ^15^ These pores lead to leakage of potassium ions from cells within minutes, leakage of cytosolic proteins such as lactate dehydrogenase (LDH) within an hour, changes in the cytoskeleton, and cytolysis.^9^, ^16^, ^17^, ^18^, ^19^ *S*. *aureus* secretes an α‐hemolysin that binds to cell membranes, with seven molecules oligomerizing to form a 1.4 nm internal diameter β‐barrel transmembrane pore, which also leads to leakage of cytosolic molecules and cytolysis.^20^, ^21^ Although cells can repair damage caused by pore‐forming toxins,^16^, ^22^, ^23^ less is known about protecting cells against these toxins.

Whilst repair encompasses replacing or fixing damaged, worn, or faulty structures, protection implies defending or guarding against attack, injury, or damage. Cell membranes most obviously need protection against pore‐forming toxins. Cell membrane cholesterol homeostasis is controlled by several mechanisms, including the action of side‐chain‐hydroxycholesterols, such as 27‐hydroxycholesterol and 25‐hydroxycholesterol.^24^, ^25^ These oxysterols act via liver X receptor transcription factors, LXRα (encoded by *NR1H3*) and LXRβ (*NR1H2*), which are both expressed in the ovary and uterus,^25^, ^26^ and by activating acetyl‐CoA acetyltransferase (ACAT) to catalyze cholesterol esterification.^27^, ^28^ However, side‐chain‐hydroxycholesterols also regulate immunity and inflammation.^4^, ^5^, ^6^, ^29^, ^30^ We therefore considered whether these oxysterols might also alter the intrinsic protection of endometrial cells against pore‐forming toxins.

Here, we tested the hypothesis that 27**‐**hydroxycholesterol and 25**‐**hydroxycholesterol help protect cells against pore‐forming toxins. We treated bovine endometrial epithelial and stromal cells with 27‐hydroxycholesterol or 25‐hydroxycholesterol, and then challenged the cells with pyolysin. We used pyolysin because *T. pyogenes* is a uterine pathogen and pyolysin damages endometrial cells.^9^, ^10^, ^18^ Cytoprotection was assessed by measuring the pyolysin‐induced leakage of potassium ions and LDH into cell supernatants, and by evaluating cell viability or changes in the cytoskeleton. We explored whether oxysterol cytoprotection was mediated via changes in cholesterol or depended on LXRs or ACAT. In addition, we examined whether the oxysterols also protected against α‐hemolysin from the endometrial pathogen, *S. aureus*.^8^ Finally, we measured the abundance of oxysterols in samples from the bovine reproductive tract.

## 2 MATERIALS AND METHODS

### 2.1 Ethics

Female genital tracts were collected after slaughter from post‐pubertal, non‐pregnant cattle that had no evidence of genital disease or microbial infection. Cattle were slaughtered during the normal work of a commercial slaughterhouse, and uteri were collected with approval from the United Kingdom Department for Environment, Food and Rural Affairs under the animal by‐products regulation (EC) No. 1069/2009 (registration number U1268379/ABP/OTHER).

### 2.2 Cell culture

Endometrial epithelial and stromal cells were isolated from uteri, cell purity confirmed, and absence of immune cell contamination verified, as described previously.^17^, ^31^ Briefly, following enzymatic digestion of the endometrium, the cell suspension was sieved through a 40‐μm mesh (pluriStrainer®, Cambridge Bioscience, Cambridge, UK) to collect stromal cells, and larger epithelial cells were then collected by back‐flushing the mesh. Cells were maintained in 75 cm^2^ flasks (Greiner Bio‐One, Gloucester, UK), at 37.5°C in humidified air containing 5% carbon dioxide, in complete medium comprising RPMI‐1640 medium (Thermo Fisher Scientific, Paisley, UK), 10% fetal bovine serum (Biosera, East Sussex, UK), and 1% antibiotic and antimycotic solution (Merck, Gillingham, UK). Culture media were replenished every 48 to 72 h, and 5 × 10^4^ passage 1 or 2 cells seeded in 1 ml/well complete medium in 24‐well tissue culture plates (TPP, Trasadingen, Switzerland), with stromal cells incubated for 24 h and epithelial cells for 48 h to achieve 70% confluency.

### 2.3 Pore‐forming toxins

The *plo* plasmid (pGS59) was a gift from Dr H Jost (University of Arizona, USA). Pyolysin, with an activity of 628,338 hemolytic units (HU)/mg protein, was generated and purified as described previously.^9^, ^32^ *S*. *aureus* α‐hemolysin (Merck) was prepared as a 0.5 mg/ml solution in deionized water, according to the manufacturer's instructions.

### 2.4 Cytoprotection experiments

The amount of pyolysin required to damage endometrial cells was determined by culturing cells for 24 h in serum‐free medium and then challenging the cells for 2 h with control serum‐free medium or medium containing the range of amounts of pyolysin specified in *Results*. Serum‐free medium was used because the serum used in the present study contained 1.12 mM cholesterol, which might confound the results by binding to pyolysin. A 2‐h challenge was used because 2 h is sufficient to cause cytolysis^9^, ^32^; and because we aimed to examine cytoprotection, whereas a longer challenge might also reflect cell repair and replication. At the end of the challenge period, pyolysin‐induced pore formation was evaluated by measuring the leakage of LDH into cell supernatants, and cytolysis was evaluated using MTT assays, as described previously.^17^

To examine whether oxysterols, steroids that regulate endometrial function, or synthetic LXR agonists protected cells against pyolysin, stromal and epithelial cells were treated in serum‐free medium containing vehicle or the range of concentrations specified in *Results* of 27‐hydroxycholesterol, 25‐hydroxycholesterol, 7β‐hydroxycholesterol or 7α‐hydroxycholesterol (all Avanti Polar Lipids, Alabama, USA), or estradiol (Merck), progesterone (Merck), hydrocortisone (Merck), or methyl‐β‐cyclodextrin (Merck) according to the manufacturers’ instructions. In the absence of data in cattle, concentrations were based on 154 ± 43 ng/ml 27‐hydroxycholesterol and 2 ± 3 ng/ml 25‐hydroxycholesterol in human plasma.^33^ Steroid concentrations spanned and exceeded physiological concentrations.^8^, ^34^ The LXR agonists T0901317 (Tocris, Abingdon, UK) and GW3965 (Tocris), were used as described previously.^35^, ^36^, ^37^ Vehicles, at a final concentration of <0.1%, were methanol for 27‐hydroxycholesterol, 25‐hydroxycholesterol, 7β‐hydroxycholesterol and 7α‐hydroxycholesterol; ethanol for hydrocortisone, progesterone, and estradiol; dimethyl sulfoxide (DMSO) for T0901317 and GW3965; and, water for methyl‐β‐cyclodextrin. Cells were treated for 24 h, based on previous cytoprotection studies^32^, ^38^; except when evaluating the duration of 27‐hydroxycholesterol treatment, where cells were treated for a range of times up to 24 h, as specified in *Results*. At the end of the treatment period, supernatants were discarded, and cells were challenged for 2 h, except where specified in *Results*, with control serum‐free medium or the selected amounts of pyolysin that caused cell damage (epithelium, 200 HU; stroma, 25 HU), without further treatment. In addition, to determine if pyolysin binds directly to treatments, pyolysin was mixed with serum‐free medium containing vehicle, 25 ng/ml 27‐hydroxycholesterol, 5 ng/ml 25‐hydroxycholesterol, 25 nM T0901317, 125 nM GW3965, or 0.5 mM methyl‐β‐cyclodextrin and then used to challenge cells for 2 h. At the end of the challenge period, cell supernatants were collected to measure the leakage of potassium or LDH, and cytolysis was evaluated using MTT assays, or by examining for changes in cells shape or cytoskeleton, as described below.^17^

To examine the role of ACAT in cytoprotection, we used the selective ACAT inhibitor Sandoz 58‐035 (SZ58‐035, Merck).^39^ Stromal and epithelial cells, 70% confluent in 24‐well culture plates, were washed twice with phosphate‐buffered saline (PBS, Merck) and treated with DMSO vehicle or 10 µM SZ58‐035 in serum‐free culture medium for 16 h, as described previously.^28^ The cells were then washed twice with PBS and treated for 24 h with 25 ng/ml 27‐hydroxycholesterol or 25 nM T0901317 in combination with vehicle or 10 µM SZ5‐035, followed by a 2‐h challenge with control medium or pyolysin. As 27‐hydroxycholesterol can activate the estrogen receptor we examined the role of the estrogen receptor using the estrogen receptor antagonists ICI 182,780 (Tocris) and MPP dihydrochloride (Tocris),^40^ as described for the ACAT inhibitor, except that we cultured the cells with DMSO vehicle or the range of concentrations specified in *Results* of ICI 182,780 or MPP dihydrochloride for 1 h, and then treated the cells with vehicle or 25 ng/ml 27‐hydroxycholesterol for 24 h in combination with vehicle, ICI 182,780 or MPP dihydrochloride, followed by the 2‐h control or pyolysin challenge. At the end of the challenge period, supernatants were collected to measure LDH, and cytolysis was evaluated by MTT assay.

To examine the role of LXRα and LXRβ in side‐chain‐hydroxycholesterol cytoprotection, we used siRNA to target *NR1H3* and *NR1H2*, respectively, as validated previously in bovine endometrial cells by >90% reduction in *NR1H3* and *NR1H2* mRNA expression.^32^ Cells were transfected with scramble siRNA (ON‐TARGETplus Non‐targeting Control Pool; Horizon discovery, Cambridge, UK) or siRNA to target *NR1H3* (sense, GCUAAAUGAUGCUGAGUUUUU; antisense, AAACUCAGCAUCAUUUAGC) or *NR1H2* (sense, AGGUGAAGGUGUCCAGUUAUU; antisense, UAACUGGACACCUUCACCU).^32^ A mixture of 20 pmol siRNA, 100 µl Opti‐MEM 1 medium and 1.5 µl Lipofectamine RNAiMAX (Thermo Fisher Scientific) were added to each well of a 24‐well plate and incubated for 20 min, and then 5 × 10^4^ stromal or epithelial cells were seeded in 900 µl/well RPMI‐1640 medium supplemented with 10% serum and cultured for 48 h. The cells were then treated with vehicle, 25 ng/ml 27‐hydroxycholesterol or 25 nM T0901317 for 24 h in serum‐free culture medium, and then challenged for 2 h with control medium or medium containing pyolysin. At the end of the challenge period, supernatants were collected to measure LDH, and cytolysis evaluated by MTT assay.

To determine if cytoprotection extended beyond cholesterol‐dependent cytolysins, after a 24‐h treatment with vehicle or 25 ng/ml 27‐hydroxycholesterol, cells were challenged for 24 h with 8 µg/well *S*. *aureus* α‐hemolysin, without further treatment. The amount and duration of α‐hemolysin challenge was based on previous work,^41^ and preliminary experiments where a shorter challenge did not cause cytolysis. At the end of the challenge period, supernatants were collected to measure the leakage of potassium or LDH, and cytolysis was evaluated using MTT assays, or by examining cells for changes in shape or the cytoskeleton.

### 2.5 MTT assay

Cell viability was assessed using the mitochondria‐dependent reduction of MTT (3‐(4,5‐dimethylthiazol‐2‐yl)‐2,5‐diphenyltetrazolium bromide, Merck), as described and validated previously for use in endometrial cells challenged with pyolysin.^9^ Briefly, cells were incubated for 2 h in 250 µl/well serum‐free culture medium containing 1 mg/ml MTT. The medium was then discarded, and cells were lysed with 300 µl DMSO (Merck). Optical density (OD_570_) was measured using a POLARstar Omega microplate reader (POLARstar Omega; BMG Labtech, Aylesbury, UK).

### 2.6 Lactate dehydrogenase

Lactate dehydrogenase was quantified using LDH‐dependent conversion of lactate to pyruvate, via reduction of β‐Nicotinamide adenine dinucleotide sodium salt (NAD+) to NADH, which is detected by NADH‐dependent reduction of a tetrazolium salt to formazan.^19^, ^42^ Briefly, a 0 to 20 nmol NADH standard curve, or 20 µl cell supernatant and 30 µl Tris‐HCL buffer pH 8.2 (Merck), were added in duplicate to a half‐area 96‐well plate (Greiner Bio‐One). An assay mixture of 54 mM sodium L‐lactate, 0.66 mM 2‐p‐iodophenyl‐3‐p‐nitrophenyl tetrazolium, 0.28 mM phenazine methosulfate, and 1.3 mM NAD+ (all Merck) was prepared in Tris‐HCL buffer pH 8.2, and 50 µl was added to each well. Optical density (OD_570_) was measured using a POLARstar Omega microplate reader before and after 30 min incubation at 37.5°C, and the difference represented the NADH generated in each well. Lactate dehydrogenase activity was defined as the amount of enzyme that catalyzed conversion of lactate to pyruvate at 37.5°C to generate 1 µmole/min NADH; the amount of LDH in each well was calculated as: LDH activity [nmole/min/ml] = (NADH × dilution factor)/(reaction time × volume of sample/well [ml]). Inter‐ and intra‐assay coefficients of variation were <4%.

### 2.7 Potassium

We measured the leakage of potassium from cells, as described previously.^17^ Cells were seeded at 1.5 × 10^5^ cells/well in 6‐well culture plates (TPP), and once 70% confluent, treated for 24 h in serum‐free medium containing vehicle, 25 ng/ml 27‐hydroxycholesterol, 5 ng/ml 25‐hydroxycholesterol, 25 nM T0901317 or 125 nM GW3965; 0.5 mM methyl‐β‐cyclodextrin was used as a control to reduce cellular cholesterol.^32^ The supernatants were discarded, the cells were washed 3 times using potassium‐free choline buffer (129 mM choline‐Cl, 0.8 mM MgCl_2_, 1.5 mM CaCl_2_, 5 mM citric acid, 5.6 mM glucose, 10 mM NH_4_Cl, 5 mM H_3_PO_4_, pH 7.4; all Merck), and then incubated in potassium‐free choline buffer containing control medium or pyolysin for 5 min, or α‐hemolysin for 15 min. Potassium was measured in cell supernatants using a Jenway PFP7 flame photometer (Cole‐Parmer, Stone, UK). Inter‐ and intra‐assay coefficients of variation were <4%.

### 2.8 Cholesterol

To determine whether the treatments altered cellular cholesterol, 70% confluent cells in 6‐well plates were treated for 24 h in serum‐free medium containing vehicle, 25 ng/ml 27‐hydroxycholesterol, 5 ng/ml 25‐hydroxycholesterol, or 0.5 mM methyl‐β‐cyclodextrin. The cells were then washed with PBS, collected in 200 µl/well cholesterol assay buffer, and cholesterol was measured using the Amplex Red Cholesterol Assay Kit (Thermo Fisher Scientific). Total protein was measured in the same samples using a DC protein assay (BioRad, Hercules, CA, United States), and cholesterol concentrations were normalized to protein, as described previously.^30^ Inter‐ and intra‐assay coefficients of variation were <6%.

### 2.9 Immunofluorescence

Phalloidin was used to stain the actin cytoskeleton, as described previously.^17^ Briefly, epithelial or stromal cells were cultured on glass cover slips in complete medium to 70% confluency; treated for 24 h in serum‐free medium containing vehicle or 25 ng/ml 27‐hydroxycholesterol; and, challenged with control medium or medium containing pyolysin for 2 h, or α‐hemolysin for 24 h. The cells were then washed with PBS, fixed with 4% paraformaldehyde, washed with PBS, and permeabilized with 0.2% Triton X‐100 (all Merck). The cells were blocked for 30 min in PBS containing 0.5% bovine serum albumin (BSA) and 0.1% Triton X‐100, and incubated for 1 h with Alexa Fluor 555‐conjugated phalloidin (Thermo Fisher Scientific). Cells were washed with 0.1% Triton X‐100 in PBS, and mounted onto microscope slides using 40,6‐diamidino2‐phenylindole to visualize cell nuclei (Vectashield with DAPI; Vector Laboratories Inc, Burlington, CA, USA). The cells were examined using an Axio Imager M1 fluorescence microscope and images were captured using an AxioCamMR3 (Zeiss). The proportion of cells that had cytoskeletal changes (cytoskeletal contraction, disrupted shape, or loss of actin fiber definition) was counted using 45 stromal or >150 epithelial cells per treatment, across 3 independent images per animal.

Filipin III from *Streptomyces filipinensis* was used to stain cholesterol,^17^ using stromal cells because they contain more cholesterol than epithelial cells.^9^ Cells on glass cover slips were treated for 24 h in serum‐free medium containing vehicle, 25 ng/ml 27‐hydroxycholesterol, 5 ng/ml 25‐hydroxycholesterol, or 0.5 mM methyl‐β‐cyclodextrin. Cells were washed with PBS, fixed with 4% paraformaldehyde, washed with PBS, and stained with 50 μg/ml filipin III for 45 min, before washing in PBS and applying 2.5% Mowiol mounting medium containing 2.5% 1,4‐diazabicyclo‐(2,2,2)‐octane (all Merck). Images were collected using a 40× objective on a Zeiss LSM710 confocal microscope, maintaining identical collection parameters. Three images were captured per treatment replicate, and fluorescence of 3 cells per image measured using Fiji.^43^

### 2.10 Western blotting

Binding of pyolysin to cells was quantified as described previously.^9^, ^11^ Stromal cells were seeded at 1.5 × 10^5^ cells/well in 6‐well plates in complete medium for 24 h, and then treated for 24 h in serum‐free medium containing vehicle, 50 nM progesterone, 0.1 nM estradiol, 10 µM hydrocortisone, 25 ng/ml 27‐hydroxycholesterol or 0.5 mM methyl‐β‐cyclodextrin, before a 2‐h challenge with control serum‐free medium or pyolysin. Cells were then washed with 300 μl ice‐cold PBS and lysed with 100 μl PhosphoSafe Extraction Reagent (Novagen, Darmstadt, Germany). Protein was extracted and 10 μg/lane separated using 10% (vol/vol) SDS‐polyacrylamide gel electrophoresis. Proteins were then transferred onto a polyvinylidene difluoride membrane (GE Healthcare, Chalfont St Giles, UK), and blocked for 1 h in Tris‐buffered saline‐Tween 20 (TBST) with 5% BSA. The membranes were then incubated overnight at 4°C with antibodies at 1:500 dilution in TBST 5% BSA for His‐pyolysin,^11^ or 1:1000 for α‐tubulin (RRID: AB_2210548; Cell Signalling, Danvers, MA, USA). Membranes were then washed 5 times in TBST and incubated for 1 h at room temperature with 1:2500 dilution anti‐rabbit IgG (RRID: AB_2099233; Cell Signalling) in TBST 5% BSA. Membranes were washed a further 5 times in TBST, and protein reactivity was visualized using enhanced chemiluminescence (Clarity Western ECL Substrate, BioRad). Membrane images were captured using a ChemiDoc XRS System (BioRad), and the average peak density of bands quantified and normalized to α‐tubulin using Fiji.^43^

To validate siRNA targeting *NR1H2* and *NR1H3*, cell lysates were stored in PhosphoSafe Extraction Reagent for western blot quantification of LXRα and LXRβ, and ABCA1, which is an LXR‐induced protein.^30^ We also examined whether treating cells with 25 ng/ml 27‐hydroxycholeterol or 25 nM T0901317 for 24 h increased ABCA1 abundance. Proteins were separated using 10% (LXR) or 7.5% (ABCA1) SDS‐polyacrylamide gel electrophoresis, as descried above. Membranes were probed with antibodies for LXRα (RRID: AB_2877144; Abcam), LXRβ (RRID: AB_776097; Abcam), ABCA1 (RRID: AB_444302; Abcam, Cambridge, UK), α‐tubulin or β‐actin (RRID: AB_306371; Abcam); incubated for 1 h with anti‐mouse IgG (RRID:AB_330924; Cell Signalling) or anti‐rabbit IgG secondary antibodies; and visualized using enhanced chemiluminescence. Average peak band density was quantified and normalized to β‐actin or α‐tubulin using Fiji.

### 2.11 Quantification of oxysterols

To quantify oxysterols in the ovary and uterus, follicular fluid was aspirated from growing and dominant ovarian follicles (4 to 8 mm and >8 mm diameter, respectively), and uterine fluid aspirated from the endometrial surface, using a 20‐gauge needle and 2 ml endotoxin‐free syringe (BD Medical, Oxford, UK). In addition, we explored whether endometrial cells also secreted oxysterols in response to lipopolysaccharide (LPS) or pyolysin, which are both major virulence factors of uterine pathogens.^8^, ^9^, ^10^, ^44^ Epithelial and stromal cells were cultured to 70% confluency in 6‐well culture plates and then incubated for 24 h in serum‐free medium, before collecting the supernatants from cells challenged for 24 h with control serum‐free medium, or a sub‐lytic amount of 5 HU pyolysin, or 1 µg/ml ultrapure LPS from *Escherichia coli* (Invivogen, Toulouse, France). The LPS also acted as a positive control because LPS stimulates the release of 25‐hydroxycholesterol from murine macrophages.^29^

Samples were extracted as described previously.^45^, ^46^ For biological samples, 100 µl was added dropwise over 5 min to 1 ml ethanol containing 2 ng [25,26,26,26,27,27,27‐^2^H_6_]24R/S‐hydroxycholesterol, 2 ng [25,26,26,26,27,27,27‐^2^H_7_]22R‐hydroxycholset‐4‐en‐3‐one, and 400 ng [25,26,26,26,27,27,27‐^2^H_7_]cholesterol (Avanti Polar Lipids) in an ultrasonic bath, and the solution was diluted to give 70% ethanol. For cell supernatants, 1 ml was added dropwise to 2.3 ml ethanol containing the same standards under sonication. Solutions were then centrifuged at 17 000 *g* for 30 min at 4°C to remove debris. Then, 200‐mg Certified Sep‐Pak C18 columns (Waters, Elstree, Herts, UK) were used to separate oxysterols and steroid acids from cholesterol, with the oxysterol‐containing fraction (SPE1‐Fr1) divided into two equal volumes (labeled A and B), dried under vacuum, and reconstituted in 100 µl isopropanol and 1 ml of 50 mM phosphate buffer (pH 7). To samples labeled “A”, we added 3 µl cholesterol oxidase from *Streptomyces* (2 mg/ml in H_2_O, 44 units/mg protein; Merck), and both A and B samples were incubated for 1 h at 37.5°C before addition of 2 ml absolute methanol and 150 µl glacial acetic acid. Finally, 150 mg [^2^H_0_]Girard P reagent (Tokyo Chemical Industry, Oxford, UK) was added to all samples and vortexed. The derivatization reaction was then allowed to occur overnight, in the dark, at room temperature, and excess derivatization reagent removed using 60‐mg Oasis HLB columns (Waters). Samples were run through the columns 3 times, and were diluted to 35% and 17.5% methanol for the second and third time, respectively. The flow through was discarded and sterols were eluted from the columns with 3 × 1 ml absolute methanol to give SPE2‐Fr1‐3 and 1 ml absolute ethanol (SPE2‐Fr4). The SPE2 fractions were analyzed on an Orbitrap Elite (Thermo Fisher Scientific) equipped with an electrospray probe, and a Dionex Ultimate 3000 LC system (Thermo Fisher Scientific) as described previously.^46^ Briefly, 2 injections were carried out per sample with 3 or more scan events per injection. One event was a high‐resolution (120 000, full width at half maximum height, at *m/z* 400) MS scan event in the Orbitrap analyzer and the rest were multi‐stage fragmentation (MSn) scan events in the linear ion trap. Quantification was performed with Xcalibur 3.0 (Thermo Fisher Scientific) by stable isotope dilution using [25,26,26,26,27,27,27‐^2^H_6_]24 R/S‐hydroxycholesterol as the internal standard; where authentic standards were unavailable, oxysterols were identified by retention time, mass and be second generation product ion (MS^3^) spectra. Peak areas were used to calculate concentrations, as described previously.^46^

### 2.12 Statistical analysis

The statistical unit was each animal used to isolate cells or to collect samples. Statistical analysis was performed using GraphPad Prism 8.4.2 (GraphPad Software, San Diego, California, USA). Data are reported as arithmetic mean (SEM), and significance attributed when *p* < .05. Comparisons between treatments or challenges were made using ANOVA followed by Dunnett, Bonferroni or Tukey post hoc test for multiple comparisons, as specified in *Results*. Correlation was evaluated using Pearson's correlation coefficient.

## 3 RESULTS

### 3.1 27‐hydroxycholesterol protected endometrial cells against pyolysin

**FIGURE 1:**
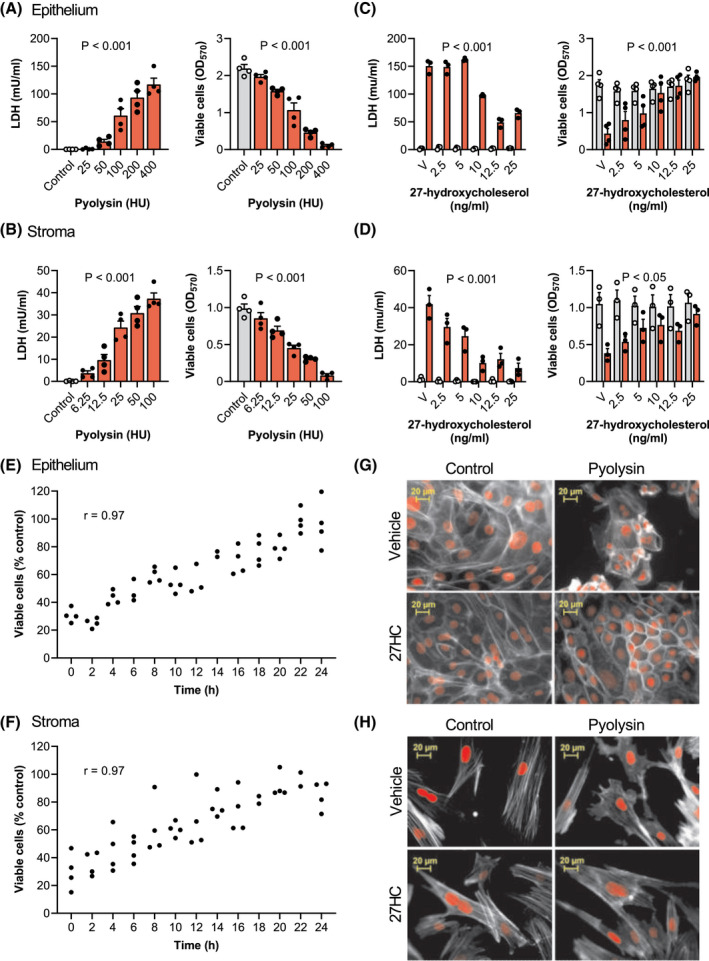
27‐hydroxycholesterol protects endometrial cells against pyolysin‐induced damage. Leakage of lactate dehydrogenase (LDH) into supernatants and viable cells determined by MTT assay for endometrial epithelial (A) and stromal cells (B) challenged for 2 h with control medium (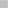) or the indicated amounts of pyolysin (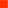). Data are presented as mean (SEM) using cells from 4 independent animals; statistical significance is determined using ANOVA. Leakage of LDH and viability of epithelial (C) and stromal cells (D) treated for 24 h with vehicle (V) or the indicated concentrations of 27‐hydroxycholesterol, and then challenged for 2 h with control medium (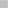) or pyolysin (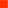, epithelium 200 HU, stroma 25 HU). Data are presented as mean (SEM) using cells from ≥3 independent animals; statistical significance is determined using two‐way ANOVA and *p* values reported for the effect of 27‐hydroxycholesterol on the pyolysin challenge. Percent viable epithelial (E) and stromal cells (F) treated for the indicated times with 25 ng/ml 27‐hydroxycholesterol, and then challenged for 2 h with control medium or pyolysin. Dots represent individual data points using cells from 4 independent animals, and Pearson correlation coefficients are reported (*r*). Fluorescent microscope images of epithelial (G) and stromal cells (H) treated for 24 h with vehicle or 25 ng/ml 27‐hydroxycholesterol (27HC), challenged for 2 h with control medium or pyolysin, and then stained with Alexa Fluor 555‐conjugated phalloidin to visualize actin (white; nuclei are red; scale bars are 20 μm). Images are representative of cells from 4 independent animals

To be able to explore cytoprotection, we first determined the amount of pyolysin needed to damage bovine endometrial cells. We evaluated pyolysin‐induced pore formation by measuring the leakage of LDH into cell supernatants, and cytolysis using MTT assays in epithelial cells (Figure 1A) and stromal cells (Figure 1B). For subsequent cytoprotection experiments, we selected pyolysin challenges that increased LDH leakage and caused cytolysis in epithelial cells (200 HU/well; *p* < .001, ANOVA and Dunnett's multiple comparison test, *n* = 4) and stromal cells (25 HU/well: *p* < .001, *n* = 4).

To examine whether side‐chain‐hydroxycholesterols are cytoprotective, cells were treated for 24 h in serum‐free medium with a range of concentrations of 27‐hydroxycholesterol, which is abundant in plasma.^33^ The cells were then challenged for 2 h with control medium or pyolysin, without further treatment. Pyolysin caused leakage of LDH, and cytolysis as expected, but treatment with 27‐hydroxycholesterol reduced pyolysin‐induced leakage of LDH and cytolysis in epithelial cells (Figure 1C) and stromal cells (Figure 1D). Specifically, 25 ng/ml 27‐hydroxycholesterol reduced epithelial and stromal cell pyolysin‐induced leakage of LDH by 56% and 82%, respectively (*p* < .001, two‐way ANOVA with Dunnett's multiple comparison test), and reduced pyolysin‐induced cytolysis from >60% to <5% and <15%, respectively (*p* < .05). The use of a control challenge (Figure 1C,D; grey bars) showed that 27‐hydroxycholesterol did not significantly alter the leakage of LDH (*p* > .99) or cell viability per se (*p* > .63). In an independent experiment, cytoprotection against pyolysin was evident after 8 h treatment of epithelial cells (*p* < .001, Figure 1E) or stromal cells (*p* = .017, Figure 1F), and for both cell types viability was correlated with the duration of 27‐hydroxycholesterol treatment (*r* ^2^ = .94, *p* < .001). As biological variation between animals might influence cytoprotection, we independently verified that 25 ng/ml 27‐hydroxycholesterol consistently reduced pyolysin‐induced cytolysis using epithelial cells from a further 6 animals and stromal cells from a further 9 animals (Supporting Information Figure S1).

To examine whether 27‐hydroxycholesterol also protected cells against changes in cell shape or the actin cytoskeleton, cells were stained with phalloidin.^17^ A 2‐h challenge with pyolysin altered the shape of epithelial and stromal cells, reduced the definition of actin fibers, and the cytoskeleton collapsed in some cells (Figure 1G,H). However, treatment with 27‐hydroxycholesterol reduced pyolysin‐induced cytoskeletal changes compared with vehicle in epithelial cells (22 ± 2% vs. 99 ± 1% cells damaged, *t*‐test, *n* = 3, *p* < .01) and stromal cells (30 ± 9% vs. 77 ± 8% cells damaged, *n* = 4, *p* < .01).

### 3.2 Oxysterols protected endometrial cells against pyolysin

**FIGURE 2:**
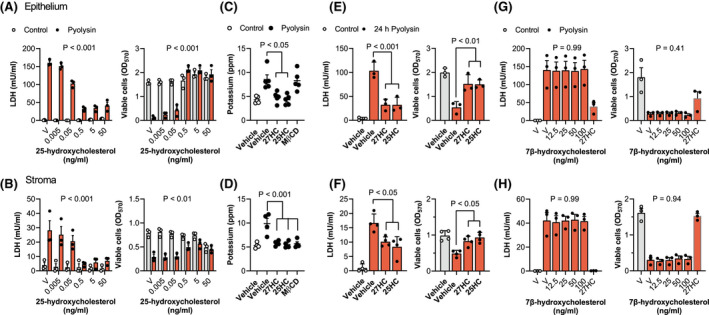
Side‐chain‐hydroxycholesterols protect endometrial cells against pyolysin. Leakage of LDH into supernatants and viable epithelial (A) and stromal cells (B) treated with vehicle (V) or the indicated concentrations of 25‐hydroxycholesterol for 24 h, and then challenged for 2 h with control medium (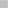) or pyolysin (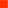, epithelium 200 HU, stroma 25 HU). Data are presented as mean (SEM) using cells from 4 independent animals; statistical significance is determined using ANOVA, and *p* values are reported for the effect of 25‐hydroxycholesterol treatment on pyolysin challenge. Potassium in supernatants of epithelial (C) and stromal cells (D) treated with vehicle, 25 ng/ml 27‐hydroxycholesterol (27HC), 5 ng/ml 25‐hydroxycholesterol (25HC) or 0.5 mM methyl‐β‐cyclodextrin (MβCD) for 24 h, and then challenged for 5 min with control medium (○) or pyolysin (●). Data are presented as mean (SEM) using cells from ≥4 independent animals; statistical significance is determined using one‐way ANOVA and Bonferroni's post hoc test. Leakage of LDH and viability of epithelial (E) and stromal cells (F) treated with vehicle, 25 ng/ml 27‐hydroxycholesterol (27HC) or 5 ng/ml 25‐hydroxycholesterol (25HC) for 24 h, and then challenged for 24 h with control medium (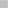) or pyolysin (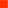). Data are presented as mean (SEM) using cells from ≥3 independent animals; statistical significance is determined using one‐way ANOVA and Bonferroni's post hoc test. Leakage of LDH into supernatants and viable epithelial (G) and stromal cells (H) treated with vehicle (V) or the indicated concentrations of 7β‐hydroxycholesterol or 25 ng/ml 27‐hydroxycholesterol (27HC) for 24 h, and then challenged for 2 h with control medium (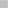) or pyolysin (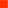, epithelium 200 HU, stroma 25 HU). Data are presented as mean (SEM) using cells from 3 independent animals; statistical significance is determined using one‐way ANOVA, and *p* values reported for the effect of 7β‐hydroxycholesterol treatment on pyolysin challenge

To explore whether cytoprotection against pyolysin extended to another side‐chain‐hydroxycholesterol, we treated endometrial cells with a range of concentrations of 25‐hydroxycholesterol, followed by a 2‐h challenge with control medium or pyolysin (Figure 2A,B). Although in the control challenge 50 ng/ml 25‐hydroxycholesterol reduced stromal cell viability by 37% (Figure 2B, grey bars, *p* < .001), lower concentrations of 25‐hydroxycholesterol did not significantly alter cell viability (Figure 2B, grey bars, *p* = .91). More importantly, treating epithelial or stromal cells for 24 h with 25‐hydroxycholesterol reduced pyolysin‐induced LDH leakage and cytolysis in epithelial (Figure 2A) and stromal cells (Figure 2B). Specifically, 5 ng/ml 25‐hydroxycholesterol reduced pyolysin‐induced LDH leakage by 84% and 79% in epithelial and stromal cells, respectively (*p* < .001, two‐way ANOVA with Dunnett's multiple comparison test), and reduced cytolysis from >60% to <5% and <25%, respectively (*p* < .01).

To evaluate whether the side‐chain‐hydroxycholesterols also protected against the initial formation of pores in cell membranes, we measured the leakage of potassium into cell supernatants 5 min after pyolysin challenge.^16^, ^17^ Before the pyolysin challenge, epithelial and stromal cells were treated with vehicle, 25 ng/ml 27‐hydroxycholesterol or 5 ng/ml 25‐hydroxycholesterol for 24 h; or with 0.5 mM methyl‐β‐cyclodextrin, as a control to reduce cellular cholesterol.^9^, ^13^ Vehicle treated cells leaked potassium after the pyolysin challenge, and methyl‐β‐cyclodextrin reduced pyolysin‐induced leakage of potassium from stromal but not epithelial cells (Figure 2C,D). However, treatment with 27‐hydroxycholesterol or 25‐hydroxycholesterol reduced pyolysin‐induced leakage of potassium from both epithelial (*p* < .05, Figure 2C) and stromal cells (*p* < .001, Figure 2D).

To test whether the cytoprotection against pyolysin persisted beyond a 2‐h challenge, we treated cells with vehicle, 25 ng/ml 27‐hydroxycholesterol or 5 ng/ml 25‐hydroxycholesterol for 24 h, and then challenged the cells with pyolysin for another 24 h, without further treatment. Both 27‐hydroxycholesterol and 25‐hydroxycholesterol reduced pyolysin‐induced leakage of LDH and cytolysis in both epithelial and stromal cells (Figure 2E,F). Taken together, these data provide evidence that 27‐hydroxycholesterol and 25‐hydroxycholesterol increased the intrinsic protection of endometrial cells against pyolysin.

One consideration was whether cytoprotection might be an artifact of pyolysin binding to side‐chain‐hydroxycholesterols. However, mixing pyolysin with 27‐hydroxycholesterol or 25‐hydroxycholesterol and then challenging untreated cells did not significantly diminish pyolysin‐induced LDH leakage or cytolysis (Supporting Information Figure S2). A second consideration was whether ring‐modified‐hydroxycholesterols might also be cytoprotective, but a range of concentrations of 7β‐hydroxycholesterol (Figure 2G,H) or 7α‐hydroxycholesterol (Supporting Information Figure S3) did not significantly protect against pyolysin‐induced leakage of LDH or cytolysis in either epithelial or stromal cells.

### 3.3 Steroid hormones do not protect endometrial cells against pyolysin

**FIGURE 3:**
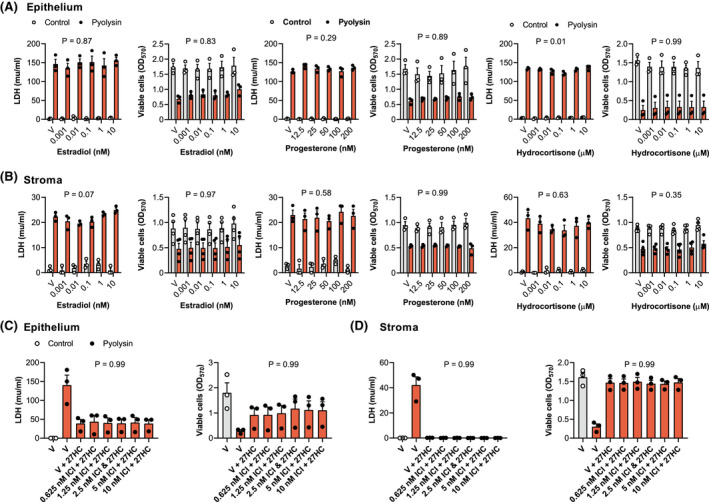
Steroid hormones do not protect endometrial cells against pyolysin. Leakage of LDH and viability of epithelial (A) and stromal cells (B) treated with vehicle (V) or the indicated concentrations of estradiol, progesterone, or hydrocortisone for 24 h, and then challenged for 2 h with control medium (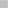) or pyolysin (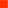, epithelium 200 HU, stroma 25 HU). Data are presented as mean (SEM) using cells from 3 or 4 independent animals; statistical significance was determined using two‐way ANOVA and *p* values reported for the effect of each treatment on the pyolysin challenge. Leakage of LDH and viability of epithelial (C) and stromal cells (D) cultured for 1 h in serum‐free medium with or without the indicated concentrations of ICI 182,780, which is an estrogen receptor antagonist, then treated with vehicle or 25 ng/ml 27‐hydroxycholesterol (in the continuing presence or absence of the antagonist) for 24 h, and then challenged for 2 h with control medium (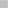) or pyolysin (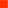). Data are presented as mean (SEM) using cells from 3 independent animals; statistical significance is determined using one‐way ANOVA and *p*‐values reported for the effect of the estrogen receptor inhibitor on the oxysterol cytoprotection

We examined whether cholesterol‐derived steroid hormones might also alter cytoprotection because they regulate endometrial function, and estradiol enhances immunity, whilst progesterone and glucocorticoids suppress immunity.^47^, ^48^, ^49^ Furthermore, although we used lower concentrations of oxysterols, micromolar concentrations of 27‐hydroxycholesterol or 25‐hydroxycholesterol can also activate the estrogen receptor.^40^, ^50^ However, there was little effect on the leakage of LDH or cytolysis after a 2‐h pyolysin challenge when epithelial or stromal cells were treated for 24 h with a range of concentrations of estradiol, progesterone, or hydrocortisone (Figure 3A,B). In addition, culturing cells with estrogen receptor antagonists ICI 182,780 or MPP dihydrochloride prior to and during treatment with 27‐hydroxycholesterol did not diminish the cytoprotection against a subsequent challenge with pyolysin (Figure 3C,D Supporting Information Figure S4).

### 3.4 Oxysterols reduced pyolysin binding and cytolysin‐accessible cholesterol in cell membranes

**FIGURE 4:**
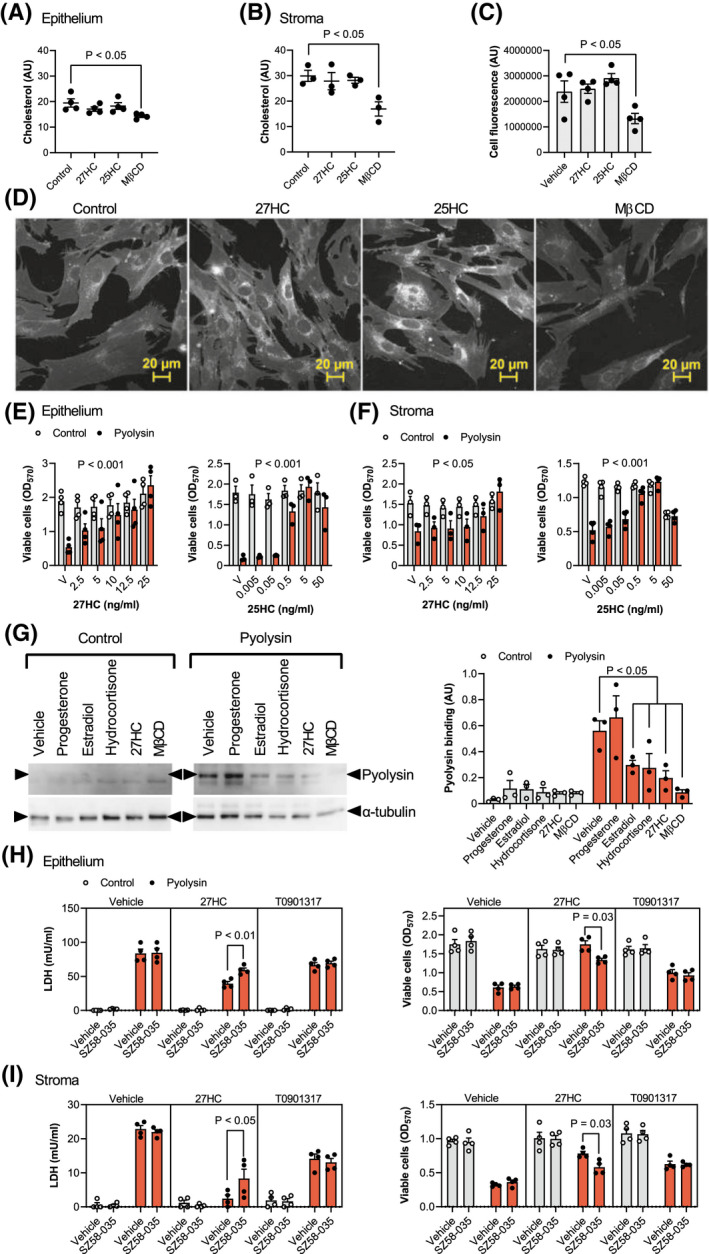
Oxysterols reduce pyolysin binding and cytolysin‐accessible cholesterol in cell membranes. Epithelial (A) and stromal cells (B to D) are cultured for 24 h in control serum‐free medium or medium containing 25 ng/ml 27‐hydroxycholesterol (27HC), 5 ng/ml 25‐hydroxycholesterol (25HC), or 0.5 mM methyl‐β‐cyclodextrin (MβCD). Cellular cholesterol is quantified and normalized to protein concentration (A, B), or total cell fluorescence was quantified (C) from confocal microscope images (D) of cells stained using filipin III to visualize cholesterol (white, scale bars are 20 µm). Data are presented as mean (SEM) using cells from 3 or 4 independent animals; statistical significance is determined using one‐way ANOVA and Dunnett's post hoc test. Viability of epithelial (E) and stromal cells (F) cultured in complete culture medium containing 10% serum and treated with vehicle (V) or the indicated concentrations of 27‐hydroxycholesterol or 25‐hydroxycholesterol for 24 h, and then challenged for 2 h with control medium (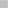) or pyolysin (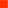, epithelium 200 HU, stroma 25 HU). Data are presented as mean (SEM) using cells from ≥3 independent animals; statistical significance is determined using ANOVA and *p* values reported for the effect of treatment on pyolysin challenge. (G) Representative western blots of pyolysin binding and α‐tubulin for stromal cells treated with vehicle, 50 nM progesterone, 0.1 nM estradiol, 10 µM hydrocortisone, 25 ng/ml 27‐hydroxycholesterol or 0.5 mM MβCD for 24 h, and then incubated with 25 HU pyolysin for 2 h. Left panel, image representative of 3 experiments; right panel, densitometry measurement of pyolysin binding normalized to α‐tubulin, with statistical significance determined using ANOVA and Dunnett's post hoc test. Leakage of LDH and viability of epithelial (H) and stromal cells (I) cultured for 16 h in serum‐free medium with or without 10 µM SZ5‐035 ACAT inhibitor, then treated with vehicle, 25 ng/ml 27‐hydroxycholesterol, or 25 nM T0901317 (in the continuing presence or absence of SZ5‐035) for 24 h, and then challenged for 2 h with control medium (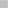) or 25 HU pyolysin (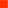). Data are presented as mean (SEM) using cells from 4 independent animals; statistical significance is determined using two‐way ANOVA and Tukey's post hoc test

We next considered potential mechanisms for side‐chain‐hydroxycholesterol cytoprotection. Reducing cellular cholesterol with methyl‐β‐cyclodextrin or cholesterol biosynthesis inhibitors is an established method to counter cholesterol‐dependent cytolysins.^9^, ^19^, ^38^ We found that treatment with 0.5 mM methyl‐β‐cyclodextrin for 24 h reduced total cellular cholesterol in epithelial cells by 26% (Figure 4A), and in stromal cells by 43% (Figure 4B). However, treatment with 27‐hydroxycholesterol or 25‐hydroxycholesterol did not significantly alter the abundance of cholesterol in epithelial cells (*p* = .37 and *p* = .81, respectively, Figure 4A), or stromal cells (*p* = .89 and *p* = .93, respectively, Figure 4B). To validate these findings, we also stained stromal cells with filipin III, which binds to unesterified cellular cholesterol.^51^ Methyl‐β‐cyclodextrin reduced filipin III fluorescence by 44% compared with vehicle (Figure 4C,D). However, although there was increased staining surrounding the nucleus, there was no significant effect of 27‐hydroxycholesterol (*p* = .98) or 25‐hydroxycholesterol (*p* = .39) on filipin III cellular fluorescence.

As cells can take up cholesterol from lipoproteins in serum,^52^ we considered the possibility that serum might alter the protective role of the oxysterols. Therefore, cells were treated with either 27‐hydroxycholesterol or 25‐hydroxycholesterol in culture medium containing 10% serum for 24 h, and then challenged with pyolysin for 2 h. As serum contains LDH,^53^ it was not possible to evaluate the leakage of LDH. However, serum did not diminish oxysterol‐mediated protection against pyolysin‐induced cytolysis in epithelial (Figure 4E) or stromal cells (Figure 4F).

As cholesterol‐dependent cytolysins specifically bind accessible cholesterol in cell membranes,^12^, ^13^, ^14^ we measured the binding of pyolysin to cells. We used stromal cells because they have more cholesterol than epithelial cells (Figure 4A,B). Treatment with methyl‐β‐cyclodextrin reduced pyolysin binding by 85%, compared with vehicle, and 27‐hydroxycholesterol reduced pyolysin binding by 65% (Figure 4G). Cytolysin‐accessible cholesterol in cell membranes can be depleted with little effect on total cellular cholesterol, by activating ACAT esterification of cholesterol with hydroxycholesterols, but not with synthetic LXR agonists.^27^, ^28^ To determine whether ACAT contributed to cytoprotection, cells were treated with the ACAT inhibitor SZ58‐035 for 16 h, followed by SZ58‐035 for a further 24 h with 27‐hydroxycholesterol or the synthetic LXR agonist T0901317, and then a 2‐h pyolysin challenge. As expected, T0901317 cytoprotection was not significantly affected by the ACAT inhibitor. However, SZ58‐035 diminished 27‐hydroxycholesterol cytoprotection against pyolysin‐induced LDH leakage and cytolysis (Figure 4H,I). Taken together, these data provide evidence that 27‐hydroxycholesterol cytoprotection against pyolysin was at least partially dependent on reducing cytolysin‐accessible cholesterol in cell membranes.

### 3.5 Oxysterol cytoprotection depends partly on liver X receptors

**FIGURE 5:**
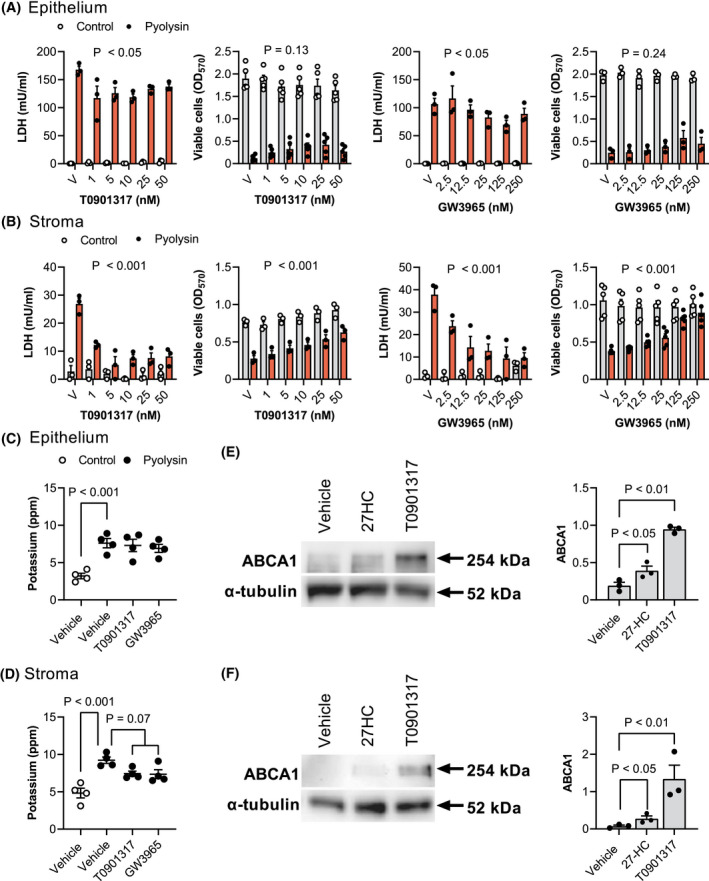
LXR agonist cytoprotection against pyolysin. Leakage of LDH and viability of epithelial (A) and stromal cells (B) treated with vehicle (V) or the indicated concentrations of LXR agonists T0901317 or GW3965 for 24 h, and then challenged for 2 h with control medium (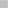) or pyolysin (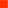, epithelium 200 HU, stroma 25 HU). Data are presented as mean (SEM) using cells from ≥3 independent animals; statistical significance is determined using two‐way ANOVA and *p* values reported for the effect of treatment on the pyolysin challenge. Potassium in supernatants from epithelial (C) and stromal cells (D) treated with vehicle, 25 nM T0901317 or 250 nM GW3965 for 24 h, and then challenged for 5 min with control medium (○) or pyolysin (●). Data are presented as mean (SEM) using cells from 4 independent animals; statistical significance is determined using ANOVA and Bonferroni's post hoc test. Representative western blot of ABCA1 and α‐tubulin for epithelial (E) and stromal cells (F) treated with vehicle, 25 ng/ml 27‐hydroxycholesterol (27HC) or 25 nM T0901317 for 24 h. Densitometry data are normalized to α‐tubulin and presented as mean (SEM) using cells from 3 independent animals; statistical significance is determined using one‐way ANOVA and *p* values reported for Dunnett's post hoc test

We next examined the role of LXRs in cytoprotection because side‐chain‐hydroxycholesterols are ligands for LXRα and LXRβ.^25^ Our first approach used synthetic ligands for both LXRα and LXRβ, T0901317 and GW3965, which are structurally unrelated to oxysterols,^25^, ^35^, ^36^, ^37^ and do not bind to pyolysin (Supporting Information Figure S2). Cells were treated for 24 h with T0901317 or GW3965, and then challenged with pyolysin for 2 h. In epithelial cells, T0901317 and GW3965 reduced pyolysin‐induced LDH leakage by up to 25% and 33%, respectively (Figure 5A, *p* < .01 and *p* < .05), but not cytolysis (*p* = .13 and *p* = .24). In stromal cells, T0901317 and GW3965 reduced pyolysin‐induced LDH leakage (Figure 5B, *p* < .001) and cytolysis (*p* < .001). Specifically, 50 nM T0901317 and 250 nM GW3965 reduced pyolysin‐induced LDH leakage from stromal cells by 70% and 75%, respectively (Figure 5B, *p* < .001). Although T0901317 or GW3965 did not significantly alter pyolysin‐induced potassium leakage from epithelial cells (Figure 5C), they tended to reduced potassium leakage from stromal cells (Figure 5D, *p* = .07). The cytoprotective concentrations of 25 ng/ml (62 nM) 27‐hydroxycholeserol, 50 nM T0901317 and 250 nM GW3965 were similar to reported EC_50_ for LXRα of 85, 85 and 190 nM, respectively.^35^, ^37^, ^54^ In addition, 25 ng/ml 27‐hydroxycholeserol and 25 nM T0901317 increased the abundance of the LXR‐dependent protein ABCA1 in epithelial and stromal cells (Figure 5E,F).

**FIGURE 6:**
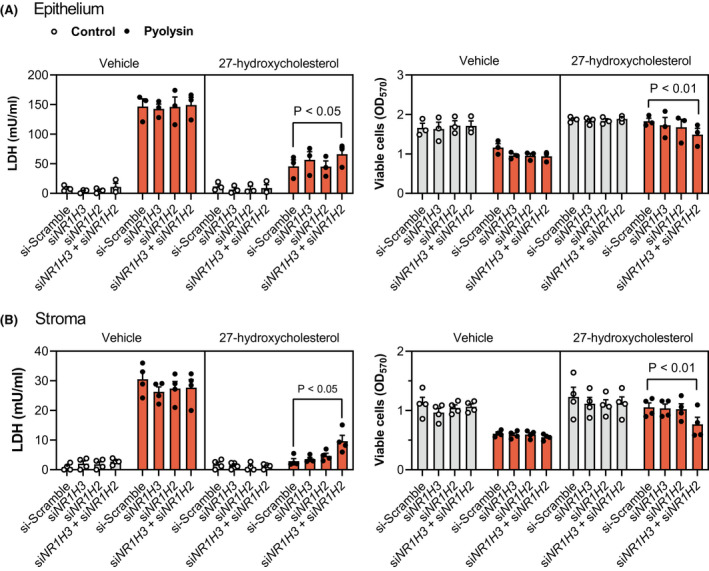
Oxysterol cytoprotection depends partly on *NR1H3* and *NR1H2*. Leakage of LDH and viability of epithelial (A) and stromal (B) cells transfected for 48 h with scramble siRNA or siRNA targeting *NR1H3, NR1H2*, or both *NR1H3* and *NR1H2*; treated with vehicle or 25 ng/ml 27‐hydroxycholesterol for 24 h; and, then challenged for 2 h with control medium (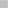) or pyolysin (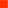, epithelium 200 HU, stroma 25 HU). Data are presented as mean (SEM) using cells from 4 independent animals; statistical significance is determined using two‐way ANOVA and Tukey's post hoc test

Our second approach was to knockdown LXRα and LXRβ by transfecting epithelial and stromal cells with previously validated siRNA targeting *NR1H3* and *NR1H2*, respectively.^32^ Additional validation in the present study showed that siRNA targeting *NR1H3* and *NR1H2* reduced LXRα and LXRβ protein abundance, and the abundance of ABCA1 (Supporting Information Figure S5). Cells were transfected with siRNA targeting *NR1H3* and *NR1H2*, independently and in combination, before treatment with 27‐hydroxycholesterol or T0901317 for 24 h, followed by a 2‐h challenge with pyolysin. Targeting both *NR1H3* and *NR1H2* diminished the 27‐hydroxycholesterol cytoprotection against pyolysin‐induced LDH leakage and cytolysis in epithelial cells (scramble vs. double knockdown, 68% vs. 56% reduction in LDH leakage, 2% vs. 21% cytolysis, Figure 6A), and in stromal cells (94% vs. 70% reduction in LDH leakage, 23% vs. 31% cytolysis, Figure 6B). Targeting both *NR1H3* and *NR1H2* also diminished T0901317 cytoprotection against pyolysin in epithelial and stromal cells (Supporting Information Figure S6). Collectively, these data provide evidence that side‐chain‐hydroxycholesterol cytoprotection against pyolysin is at least partially dependent on LXRα and LXRβ.

### 3.6 Oxysterols protected cells against α‐hemolysin

**FIGURE 7:**
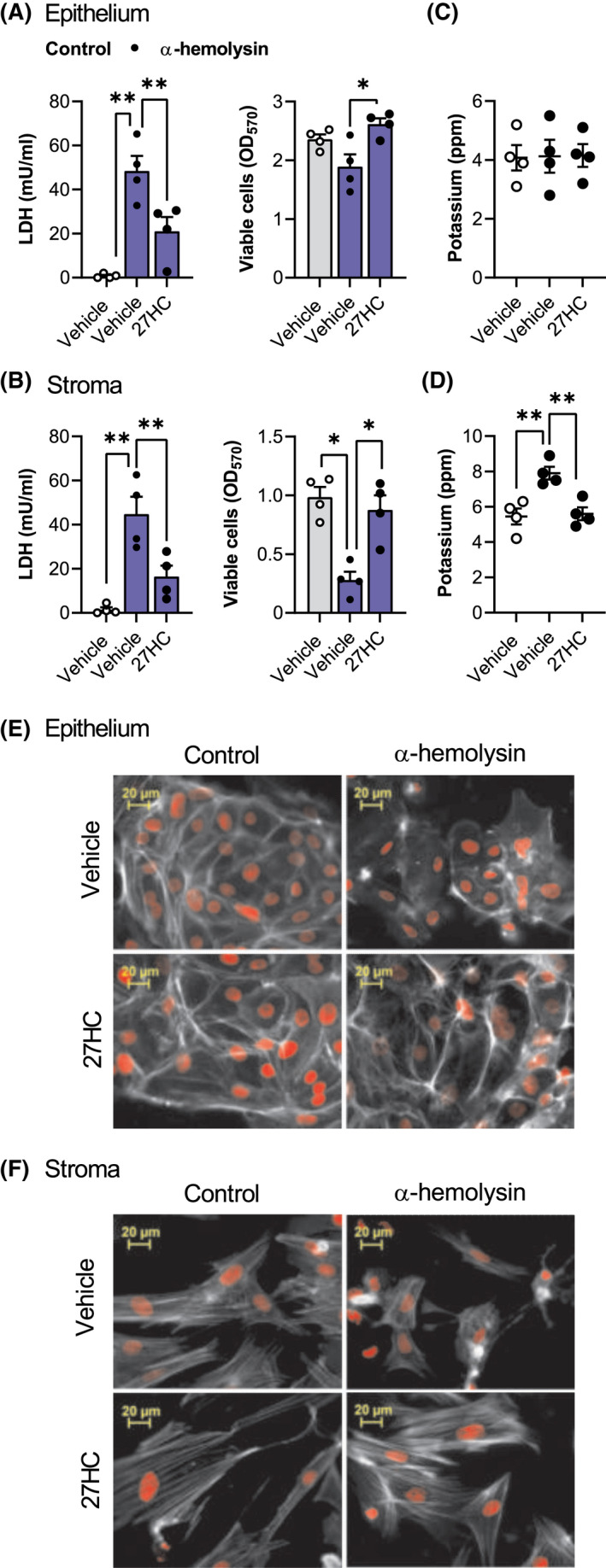
Side‐chain‐hydroxycholesterols protect endometrial cells against *Staphylococcus aureus* α‐hemolysin. Leakage of LDH and viability of epithelial (A) and stromal (B) cells treated with vehicle or 25 ng/ml 27‐hydroxycholesterol (27HC) for 24 h, and then challenged for 24 h with control medium (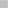) or 8 µg α‐hemolysin (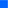). Data are presented as mean (SEM) using cells from 4 independent animals; statistical significance is determined using ANOVA and Dunnett's multiple comparison post hoc test, *p < .05; **p < .01. Potassium in supernatants from epithelial (C) and stromal cells (D) treated with vehicle or 25 ng/ml 27‐hydroxycholesterol for 24 h, and then challenged for 15 min with control medium (○) or 8 µg α‐hemolysin (●). Data are presented as mean (SEM) using cells from 4 independent animals; statistical significance is determined using ANOVA and Dunnett's post hoc test. Fluorescent microscope images of epithelial (E) and stromal cells (F) treated with vehicle or 25 ng/ml 27‐hydroxycholesterol (27HC) for 24 h, challenged for 24 h with control medium or 8 µg α‐hemolysin, and then stained with Alexa Fluor 555‐conjugated phalloidin to visualize actin (white; nuclei are red; scale bars are 20 μm). Images are representative of cells from 3 animals

To determine whether cytoprotection extended beyond pyolysin, we treated cells with vehicle or 25 ng/ml 27‐hydroxycholesterol, and then challenged the cells with *S. aureus* α‐hemolysin for 24 h, without further treatment. The α‐hemolysin challenge induced leakage of LDH (Figure 7A,B), reduced stromal cell viability (Figure 7B) and tended to reduce epithelial cell viability (Figure 7A, *p* = .1). However, treating epithelial or stromal cells with 27‐hydroxycholesterol reduced α‐hemolysin‐induced leakage of LDH and cytolysis. Although potassium leakage was not detected from epithelial cells following a 15‐min α‐hemolysin challenge (Figure 7C), 27‐hydroxycholesterol reduced α‐hemolysin‐induced leakage of potassium from stromal cells (Figure 7D). Treatment with 27‐hydroxycholesterol also reduced α‐hemolysin‐induced cytoskeletal changes in epithelial cells (Figure 7E; 44 ± 3% vs. 90 ± 3% cells damaged, *n* = 3, *p* < .01) and stromal cells (Figure 7F; 9 ± 1% vs. 62 ± 6% cells damaged, *n* = 3, *p* < .01). Together, these data provide evidence for 27‐hydroxycholesterol cytoprotection of endometrial cells against *S. aureus* α‐hemolysin.

### 3.7 Endometrial cells released oxysterols

**FIGURE 8:**
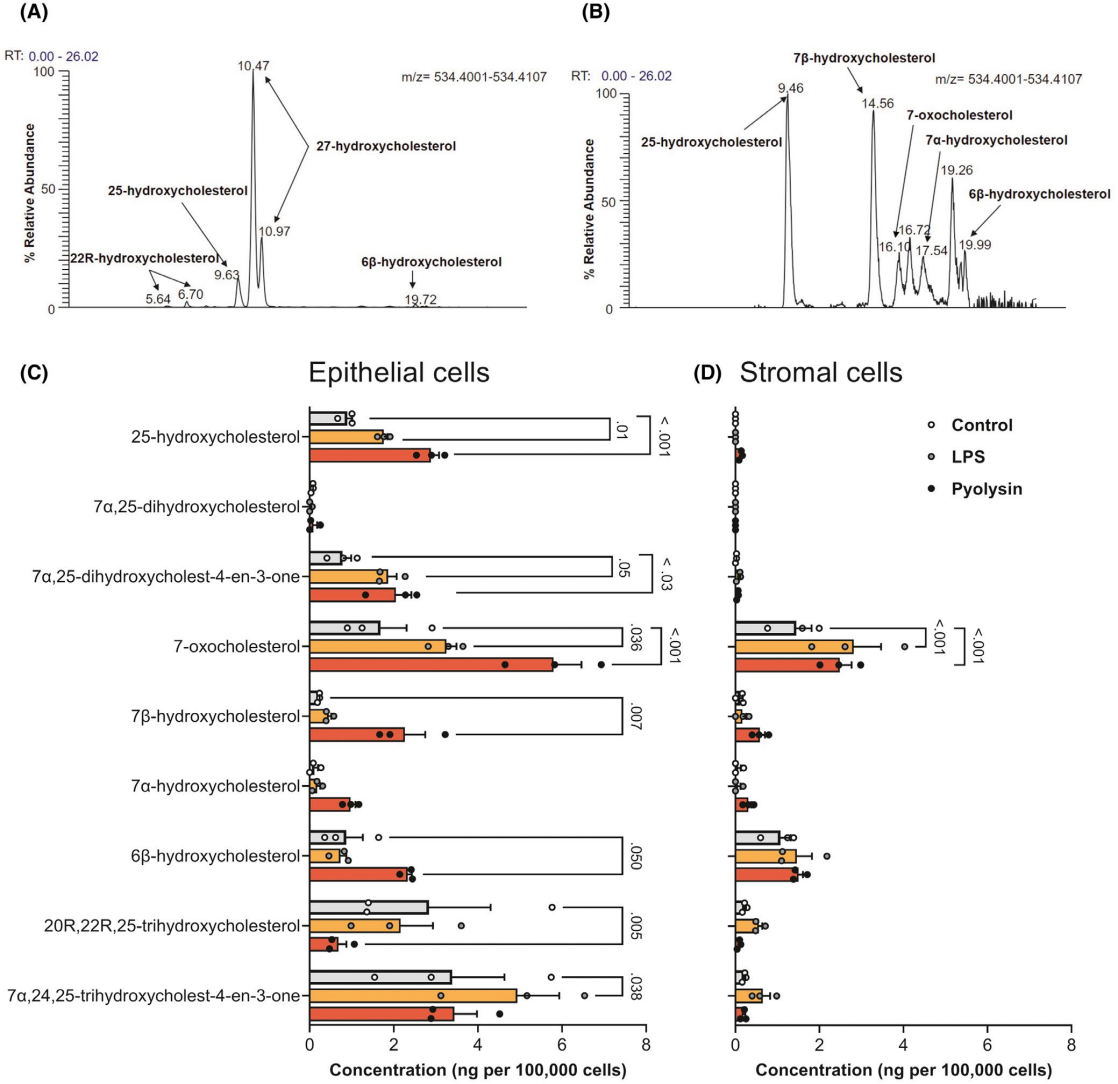
Oxysterols in the bovine reproductive tract. Chromatographic separation (*m/z* 534.4054 ± 5 ppm) of indicated oxysterols from (A) dominant ovarian follicular fluid sample derivatized with [^2^H_0_] Girard P reagent, and (B) supernatant of epithelial cells challenged for 24 h with 5 HU pyolysin. Concentrations of selected oxysterols in supernatants from epithelial (C) and stromal cells (D) challenged for 24 h with control medium (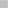), or medium containing 1 µg/ml LPS (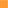) or 5 HU pyolysin (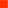). Data are presented as mean (SEM) using cells from 3 independent animals; statistical significance is determined using two‐way ANOVA and Dunnett's multiple comparison post hoc test, with *p*‐values reported for difference from control

To better understand the biological relevance of oxysterols in the bovine reproductive tract, we used mass spectrometry and found a range of oxysterols in uterine and ovarian follicular fluid (Table 1, Figure 8A). Notably, 27‐hydroxycholesterol and 25‐hydroxycholesterol were detected in uterine fluid, as well as the metabolite of 25‐hydroxycholesterol, 7α,25‐hydroxycholesterol. As ovarian steroids regulate endometrial function,^47^ it was interesting that there were higher concentrations of oxysterols in ovarian follicular fluid collected from both growing and dominant follicles than in uterine fluid.

**TABLE 1:** Oxysterol concentrations in biological samples

Oxysterol	Uterus (ng/ml)	Growing follicle (ng/ml)	Dominant follicle (ng/ml)
22R‐hydroxycholesterol	0.2 ± 0.2	2.3 ± 0.8	2.4 ± 1.5
24S‐hydroxycholesterol	0.2 ± 0.2	0.2 ± 0.1	0.5 ± 0.1
25‐hydroxycholesterol	3.9 ± 0.6	13.4 ± 10.5	26.8 ± 11.4
25‐hydroxycholest‐4‐en‐3‐one	0.0 ± 0.0	0.1 ± 0.1	2.1 ± 1.8
27‐hydroxycholesterol	19.9 ± 4.9	104.3 ± 69.7	244.6 ± 99.5
27‐hydroxycholest‐4‐en‐3‐one	0.0 ± 0.0	0.5 ± 0.2	6.8 ± 5.2
7β‐hydroxycholesterol	0.8 ± 0.8	0.4 ± 0.2	0.4 ± 0.2
7α‐hydroxycholesterol	8.3 ± 4.9	0.9 ± 0.6	1.1 ± 0.0
7α‐hydroxycholest‐4‐en‐3‐one	17.0 ± 8.4	0.2 ± 0.1	1.3 ± 1.1
6β‐hydroxycholesterol^a^	7.1 ± 0.9	2.3 ± 0.6	2.2 ± 0.2
6β‐hydroxycholest‐4‐en‐3‐one^a^	0.8 ± 0.5	0.2 ± 0.1	0.9 ± 0.6
7‐oxocholesterol	13.2 ± 1.5	2.8 ± 1.0	6.8 ± 5.1
24,25‐epoxycholesterol	0.6 ± 0.6	7.5 ± 4.9	9.3 ± 7.3
24,25‐epoxycholest‐4‐en‐3‐one	0.0 ± 0.0	0.3 ± 0.2	6.6 ± 5.3
24,27‐dihydroxycholesterol	0.5 ± 0.5	5.8 ± 3.0	4.8 ± 3.0
24,27‐dihydroxycholest‐4‐en‐3‐one	0.0 ± 0.0	0.3 ± 0.2	2.6 ± 2.3
7α,25‐dihydroxycholesterol	1.3 ± 0.7	0.7 ± 0.4	2.0 ± 1.5
7α,25‐dihydroxycholest‐4‐en‐3‐one	1.5 ± 0.4	1.5 ± 0.5	16.7 ± 12.6
7α,27‐dihydroxycholesterol	0.0 ± 0.0	0.2 ± 0.2	0.0 ± 0.0
7α,27‐dihydroxycholest‐4‐en‐3‐one	2.3 ± 0.7	1.3 ± 0.2	13.8 ± 6.4
3β,7β‐dihydroxycholest‐5‐en‐26‐oic acid	0.0 ± 0.0	2.3 ± 0.2	2.0 ± 1.0
7β‐hydroxy‐3‐oxocholest‐4‐en‐26‐oic acid	0.0 ± 0.0	0.3 ± 0.17	0.5 ± 0.2
3β,7α‐dihydroxycholest‐5‐en‐26‐oic acid	4.9 ± 2.5	13.2 ± 6.9	6.5 ± 4.2
7α‐hydroxy‐3‐oxocholest‐4‐en‐26‐oic acid	81.3 ± 20.0	76.8 ± 21.0	258.9 ± 136.8
3β,22R‐dihydroxycholest‐5‐en‐26‐oic acid	0.0 ± 0.0	23.5 ± 14.7	23.9 ± 13.5
22R‐hydroxy‐3‐oxocholest‐4‐en‐26‐oic acid	0.0 ± 0.0	3.0 ± 1.6	11.1 ± 4.7
20R,22R,25‐trihydroxycholesterol	0.9 ± 0.9	10.7 ± 5.5	2.4 ± 1.8
20R,22R,25‐trihydroxycholest‐4‐en‐3‐one	1.1 ± 0.8	2.4 ± 0.7	39.5 ± 21.3
7α,24,25‐trihydroxycholest‐4‐en‐3‐one	4.8 ± 2.5	4.3 ± 1.3	20.5 ± 13.2
3β‐hydroxycholest‐5‐enoic acid	12.7 ± 6.8	283.3 ± 249.4	295.8 ± 118.4
3‐oxocholest‐4‐enoic acid	4.4 ± 3.1	20.8 ± 12.9	11.1 ± 4.7
3β,7α,24‐trihydroxycholest‐5‐en‐26‐oic acid	0.0 ± 0.0	0.57 ± 0.57	0.0 ± 0.0
7α,24‐dihydroxy‐3‐oxocholest‐4‐en‐26‐oic acid	3.6 ± 1.8	5.1 ± 0.2	16.4 ± 6.8
3β,7α,25‐trihydroxycholest‐5‐en‐26‐oic acid	0.0 ± 0.0	0.8 ± 0.6	1.5 ± 0.9
7α,25‐dihydroxy‐3‐oxocholest‐4‐en‐26‐oic acid	2.5 ± 1.4	3.1 ± 1.1	12.3 ± 6.3

Note

Concentrations of oxysterols in samples of uterine fluid or follicular fluid from growing (4 to 8 mm diameter) or dominant (>8 mm diameter) ovarian follicles (data are reported as mean ± SEM, *n* = 3 per sample).

^a^ Dehydration products of cholestane‐3β,5α,6β‐triol.

We next considered whether endometrial cells might also secrete oxysterols in response to a sub‐lytic amount of 5 HU pyolysin for 24 h. We used 1 µg/ml LPS as a control because it is a major virulence factor of uterine pathogens^44^; and, because LPS stimulates the release of 25‐hydroxycholesterol from murine macrophages.^29^ Few oxysterols were detectable in serum‐free culture medium (Table 2). However, epithelial cell supernatants contained several oxysterols, including 25‐hydroxycholesterol (Figure 8B,C), and concentrations of 25‐hydroxycholesterol increased by 220% following pyolysin challenge. The supernatants of stromal or epithelial cells had no detectable 27‐hydroxycholesterol, and 25‐hydroxycholesterol was barely detectable in stromal cells (Figure 8D). Together these data provide evidence for oxysterols in the reproductive tract, and the release of 25‐hydroxycholesterol from epithelial cells challenged with pyolysin.

**TABLE 2:** Oxysterol concentrations in culture medium

Oxysterol	ng/ml
6β‐hydroxycholesterol	0.39
6β‐hydroxycholest‐4‐en‐3‐one	0.24
7‐oxocholesterol	1.15

Note

Concentrations of oxysterols detected in serum‐free culture medium.

## 4 DISCUSSION

In this study we discovered that both 27‐hydroxycholesterol and 25‐hydroxycholesterol increased the intrinsic protection of bovine endometrial cells against pore‐forming toxins from uterine pathogens. Treating epithelial or stromal cells with the side‐chain‐hydroxycholesterols reduced the formation of pyolysin‐induced cell membrane pores and protected cells against damage. This oxysterol cytoprotection was partly dependent on ACAT reducing cytolysin‐accessible cholesterol in the cell membrane and on LXRs. Interestingly, oxysterols were also found in the reproductive tract, and there was increased release of 25‐hydroxycholesterol from epithelial cells in response to pyolysin. We suggest that side‐chain‐hydroxycholesterols help protect the endometrium against pore‐forming toxins from pathogenic bacteria.

Pyolysin formed pores in endometrial cell membranes as determined by the leakage of potassium and LDH, caused cell damage as determined by changes in cell shape and the cytoskeleton, and reduced cell viability. These effects are typical of cholesterol‐dependent cytolysins, which are the most common pore‐forming toxins secreted by pathogenic bacteria.^7^, ^15^, ^16^, ^22^ Endometrial cells were also damaged by α‐hemolysin, but the cells required a longer challenge than for pyolysin, which might reflect the ten‐fold smaller pore diameter of α‐hemolysin than pyolysin.^7^, ^15^, ^20^ Epithelial cells were less susceptible than stromal cells to damage by either toxin, which supports the concept of a resilient epithelium helping tissues to tolerate pathogens.^3^, ^9^ However, damage to the epithelium during and after parturition increases the risk of developing uterine disease once the underlying sensitive stromal cells are exposed to *T. pyogenes* or *S*. *aureus*.

Our most striking finding was that the side‐chain‐hydroxycholesterols 27‐hydroxycholesterol or 25‐hydroxycholesterol, but not the ring‐modified‐hydroxycholesterols 7β‐hydroxycholesterol or 7α‐hydroxycholesterol, protected endometrial epithelial and stromal cells against cytolysis in the face of pyolysin challenges that normally caused >50% cytolysis. Treatment with 25 ng/ml 27‐hydroxycholesterol or 5 ng/ml 25‐hydroxycholesterol also reduced the leakage of potassium and LDH, and limited pyolysin‐induced changes in cell shape and the actin cytoskeleton. Cytoprotection was effective even when cells were treated in medium containing serum, and protection against the pyolysin challenge persisted for at least 24 h. Our findings support recent observations that murine macrophages stimulated with interferon are protected against cholesterol‐dependent cytolysins perfringolysin O, streptolysin O and anthrolysin O, via an oxysterol‐dependent mechanism.^55^ Furthermore, injection of 25‐hydroxycholesterol into the skin of mice reduced tissue damage caused by anthrolysin O.^55^ Although we found that 27‐hydroxycholesterol also protected endometrial cells against α‐hemolysin, cytoprotection against other classes of pore‐forming toxins warrants further study.

The physiological or pathological roles of oxysterols in the uterus have not been considered previously, and sources of these oxysterols is yet to be resolved. While much remains to be discovered about oxysterols in the female genital tract of cattle, we found both 27‐hydroxycholesterol and 25‐hydroxycholesterol in uterine fluid and ovarian follicular fluid, and endometrial epithelial cells also released 25‐hydroxycholesterol. Notably, endometrial cells did not release 27‐hydroxycholesterol, which indicates that endometrial cells were not the origin of this oxysterol in the biological fluids. However, the concentrations of 27‐hydroxycholesterol and 25‐hydroxycholesterol we measured in uterine fluid were similar to the concentrations we used to treat endometrial cells. Although the innate immune response to LPS triggers secretion of 25‐hydroxycholesterol from macrophages,^6^, ^29^ we unexpectedly found that pyolysin also stimulated the secretion of 25‐hydroxycholesterol. We suggest that epithelial cells might release 25‐hydroxycholesterol to help protect stromal cells against pore‐forming toxins. This oxysterol cytoprotection would add to the existing role for oxysterols in regulating immunity to pathogens.^4^, ^5^, ^6^ Steroids also modulate defenses against pathogens in the uterus, with estradiol reducing the risk of disease, and progesterone and hydrocortisone increasing the risk of disease.^47^, ^48^, ^49^ However, in the present study, estradiol, progesterone, and hydrocortisone did not alter pyolysin‐induced leakage of LDH or cytolysis. There was also no evidence that 27‐hydroxycholesterol cytoprotection was mediated via the estrogen receptor. Instead, side‐chain‐hydroxycholesterols may have a paracrine role to enhance cell‐intrinsic protection against pore‐forming toxins. This provides an additional example of a mechanism that tissues use to tolerate the presence of pathogens.^3^

Oxysterols regulate cholesterol homeostasis, increase cholesterol esterification, modify cell membranes and activate LXRs.^5^, ^24^, ^25^, ^27^, ^28^ Cell membranes contain three pools of cholesterol: an essential pool, a sphingomyelin‐bound pool, and a pool of cholesterol accessible to cholesterol‐dependent cytolysins.^13^, ^14^ Only cytolysin‐accessible cholesterol in cell membranes is “labile”, and small changes in cholesterol synthesis, uptake, efflux or metabolism markedly alter cholesterol‐dependent cytolysins binding to cells.^13^, ^14^, ^28^ In the present study, 27‐hydroxycholesterol reduced pyolysin binding to cells, even though the oxysterols did not significantly alter total cellular cholesterol. Our findings agree with recent observations that 27‐hydroxycholesterol and 25‐hydroxycholesterol reduce binding of anthrolysin O to cells, without reducing total cellular cholesterol.^28^, ^55^ The oxysterols were thought to have reduced cytolysin‐accessible cholesterol in the cell membrane by limiting cholesterol biosynthesis or activating ACAT esterification of cholesterol.^28^, ^55^ In the present study, an ACAT inhibitor also diminished oxysterol cytoprotection against pyolysin. However, we additionally found that targeting both *NR1H3* and *NRIH2* with siRNA diminished oxysterol cytoprotection against pyolysin. Furthermore, synthetic LXR ligands reduced pyolysin‐induced leakage of LDH from stromal cells by ≥70%; almost as effectively as the ≥80% reduction by 27‐hydroxycholesterol or 25‐hydroxycholesterol. Our observations that oxysterol cytoprotection in bovine cells was partially mediated via ACAT supports similar observations in mice.^28,55^

Although side‐chain‐hydroxycholesterol cytoprotection partially depended on ACAT and LXRs, they likely provide cytoprotection via more than just these mechanisms. Oxysterol‐accelerated degradation of 3‐hydroxy‐3‐methylglutaryl CoA reductase,^56^ which is the rate‐limiting enzyme in cholesterol biosynthesis, might also contribute to reductions in cytolysin‐accessible cholesterol. Consistent with this potential mechanism, we have shown previously that statins or siRNA targeting 3‐hydroxy‐3‐methylglutaryl CoA reductase partially protects bovine endometrial epithelial cells against pyolysin.^57^ The action of oxysterols might also be integrated with the multiple complementary cell repair mechanisms activated by cytolysins.^9^, ^16^, ^22^, ^23^, ^31^, ^58^ For example, oxysterols stimulate increased cytosolic calcium, which could activate protective repair mechanisms such as endocytosis, membrane blebbing, caspase activity, and the unfolded protein response.^16^, ^23^, ^58^, ^59^ Further work is required to establish whether these mechanisms contribute to oxysterol cytoprotection. As side‐chain‐hydroxycholesterols only differ from cholesterol by one hydroxyl group, another possibility was that they might bind to cholesterol‐dependent cytolysins to neutralize their activity. However, the cholesterol‐dependent cytolysin perfringolysin O did not bind to 25‐hydroxycholesterol in liposomes, even with concentrations up to 60 mol% 25‐hydroxycholesterol.^12^ In the present study there was also no evidence of pyolysin binding oxysterols, because the oxysterol treatment and pyolysin challenge were independent; cytoprotection correlated with the duration of oxysterol treatment; mixing pyolysin with oxysterols did not diminish cytolysis; and, oxysterols protected against α‐hemolysin, which is less dependent on cytolysin‐accessible cholesterol in the cell membrane than pyolysin.^7^, ^21^

In conclusion, we found that side‐chain‐hydroxycholesterols increased endometrial cell‐intrinsic protection against pore‐forming toxins. It was striking that both 27‐hydroxycholesterol and 25‐hydroxycholesterol protected epithelial and stromal cells against both pyolysin and α‐hemolysin. The oxysterol cytoprotection against pyolysin included ACAT and LXR dependent mechanisms. We also found the side‐chain‐hydroxycholesterols in uterine and ovarian fluids, and that epithelial cells released 25‐hydroxycholesterol in response to pyolysin. Collectively, our findings imply that side‐chain‐hydroxycholesterols help protect bovine endometrial cells against pore‐forming toxins and defend the endometrium against pathogenic bacteria.

## DISCLOSURES

The authors have stated that there are no conflicts of interest in connection with this article.

## AUTHOR CONTRIBUTIONS

Iain M. Sheldon, John J. Bromfield, and Thomas J. R. Ormsby designed research. Thomas J. R. Ormsby and Sian E. Owens performed research. William J. Griffiths, Yuqin Wang, Daphne Davies, and Anthony D. Horlock contributed new reagents or analytic tools. Iain M. Sheldon, Thomas J. R. Ormsby and William J. Griffiths analyzed data. Iain M. Sheldon, Thomas J. R. Ormsby, William J. Griffiths, James G. Cronin, and John J. Bromfield wrote the paper.

## Supporting information

Fig S1

Fig S2

Fig S3

Fig S4

Fig S5

Fig S6
