# Supplementary material for: Oxysterols protect bovine endometrial cells against pore‐forming toxins from pathogenic bacteria
Source: FASEB J. 2021 Sep 27;35(10):e21889. doi: 10.1096/fj.202100036R (PMC9272411; doi:10.1096/fj.202100036R)
Supplement: Supplementary file 1 — Fig S1 [file FSB2-35-e21889-s003.pdf]

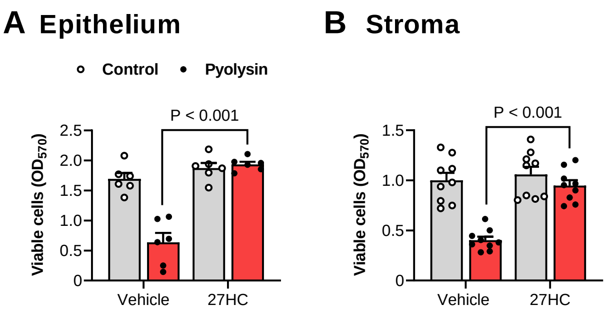

Supplemental Figure 1. 27-hydroxycholesterol cytoprotection against pyolysin

Epithelial (A) and stromal cells (B) were cultured for 24 hours in serum-free medium containing vehicle or 25 ng/ml 27-hydroxycholesterol (27HC), and then challenged for 2 hours with control medium (■) or pyolysin (■, epithelium 200 HU, stroma 25 HU). Cell viability was determined by MTT assay. Data are presented as mean (SEM) using cells from 6 independent animals for epithelial cells and 9 animals for stromal cells; statistical significance was determined using two-way ANOVA and Bonferroni's post hoc test.
