# Supplementary material for: Oxysterols protect bovine endometrial cells against pore‐forming toxins from pathogenic bacteria
Source: FASEB J. 2021 Sep 27;35(10):e21889. doi: 10.1096/fj.202100036R (PMC9272411; doi:10.1096/fj.202100036R)
Supplement: Supplementary file 2 — Fig S2 [file FSB2-35-e21889-s001.pdf]

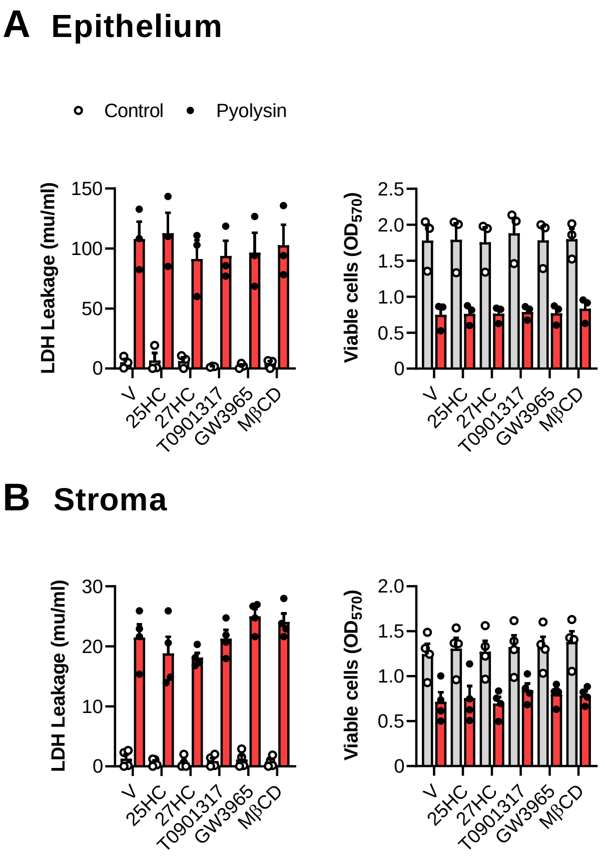

Supplemental Figure 2. Mixing pyolysin with treatments did not prevent cytolysis

Epithelial (A) and stromal cells (B) were cultured for 24 hours in serum-free medium and then challenged for 2 hours with control serum-free medium (■) or pyolysin (■, epithelium 200 HU, stroma 25 HU), which had been mixed with vehicle, 5 ng/ml 25-hydroxycholesterol (25HC), 25 ng/ml 27-hydroxycholesterol (27HC), 25 nM T0901317, 125 nM GW3965, or 0.5 mM MβCD. The leakage of LDH into cell supernatants was measured, and cell viability was determined by MTT assay. Data are presented as mean (SEM) using cells from 4 independent animals; statistical significance was determined using two-way ANOVA, but treatments did not differ significantly from vehicle for pyolysin challenge.
