# Supplementary material for: Oxysterols protect bovine endometrial cells against pore‐forming toxins from pathogenic bacteria
Source: FASEB J. 2021 Sep 27;35(10):e21889. doi: 10.1096/fj.202100036R (PMC9272411; doi:10.1096/fj.202100036R)
Supplement: Supplementary file 3 — Fig S3 [file FSB2-35-e21889-s002.pdf]

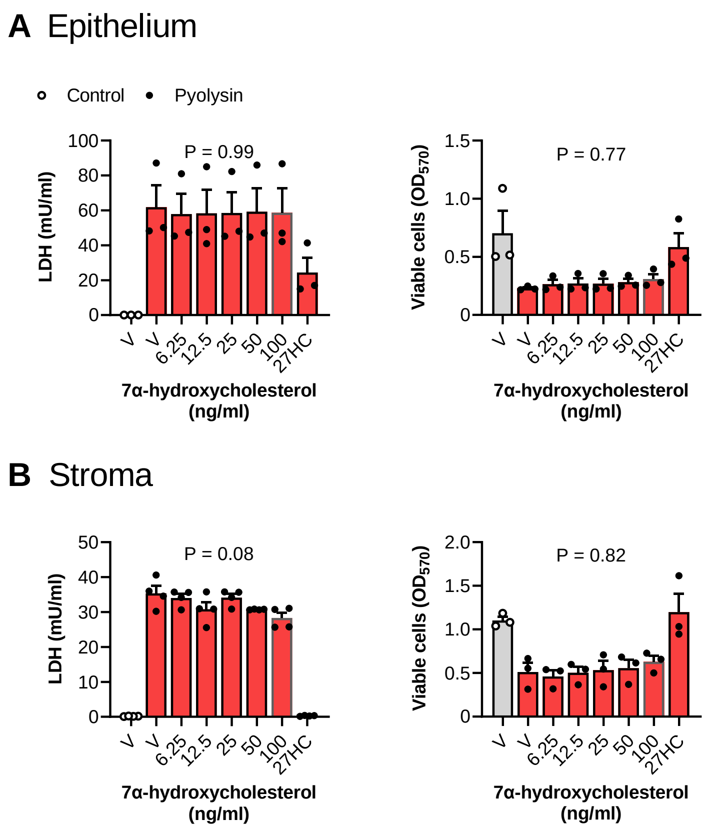

Supplemental Figure 3. 7α-hydroxycholesterol was not cytoprotective against pyolysin

Epithelial (A) and stromal cells (B) were cultured for 24 hours in serum-free medium containing vehicle, the indicated concentration of 7α-hydroxycholesterol or 25 ng/ml 27-hydroxycholesterol (27HC), and then challenged for 2 hours with control medium (■) or pyolysin (■, epithelium 200 HU, stroma 25 HU). Cell viability was determined by MTT assay. Data are presented as mean (SEM) using cells from 3 or 4 independent animals; statistical significance was determined using one-way ANOVA, and P values reported for the effect of 7α-hydroxycholesterol treatment on pyolysin challenge.
