# Supplementary material for: Oxysterols protect bovine endometrial cells against pore‐forming toxins from pathogenic bacteria
Source: FASEB J. 2021 Sep 27;35(10):e21889. doi: 10.1096/fj.202100036R (PMC9272411; doi:10.1096/fj.202100036R)
Supplement: Supplementary file 4 — Fig S4 [file FSB2-35-e21889-s005.pdf]

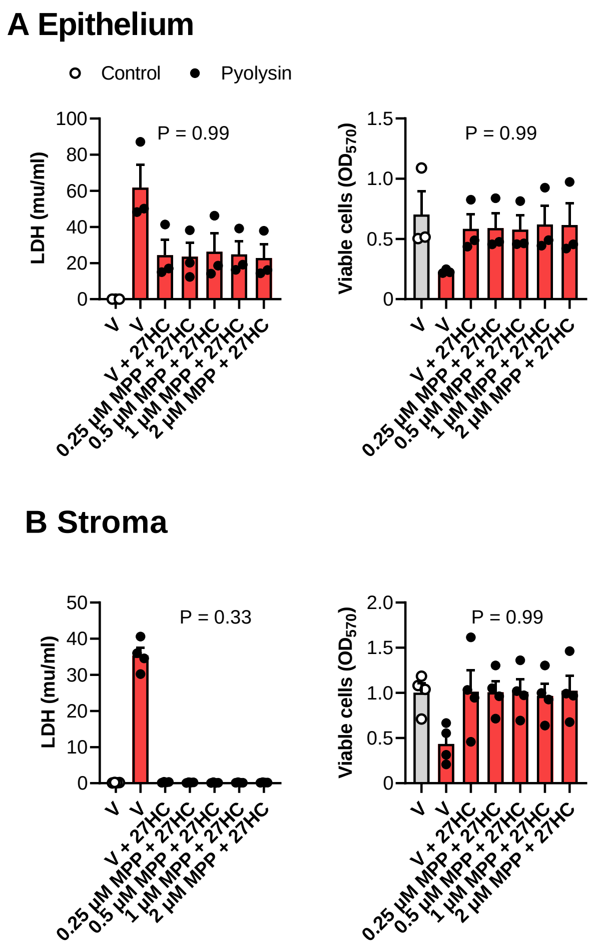

Supplemental Figure 4. Estradiol receptor antagonist MPP dihydrochloride did not diminish 27-hydroxycholesterol cytoprotection against pyolysin

Leakage of LDH and viability of epithelial (A) and stromal cells (B) cultured for 1 hour in serum-free medium with or without the indicated concentrations of the estradiol receptor alpha antagonist MPP dihydrochloride (1,3-*Bis*(4-hydroxyphenyl)-4-methyl-5-[4-(2-piperidinylethoxy)phenol]-1*H*-pyrazole dihydrochloride), then treated with vehicle (V) or 25 ng/ml 27-hydroxycholesterol (27HC) in the continuing presence or absence of the antagonist for 24 hours, and then challenged for 2 hours with control medium (■) or pyolysin (■, epithelium 200 HU, stroma 25 HU). Data are presented as mean (SEM) using cells from 3 or 4 independent animals; statistical significance was determined using one-way ANOVA and P-values reported for the effect of the estradiol receptor antagonist on the oxysterol cytoprotection.
