# Supplementary material for: Oxysterols protect bovine endometrial cells against pore‐forming toxins from pathogenic bacteria
Source: FASEB J. 2021 Sep 27;35(10):e21889. doi: 10.1096/fj.202100036R (PMC9272411; doi:10.1096/fj.202100036R)
Supplement: Supplementary file 5 — Fig S5 [file FSB2-35-e21889-s004.pdf]

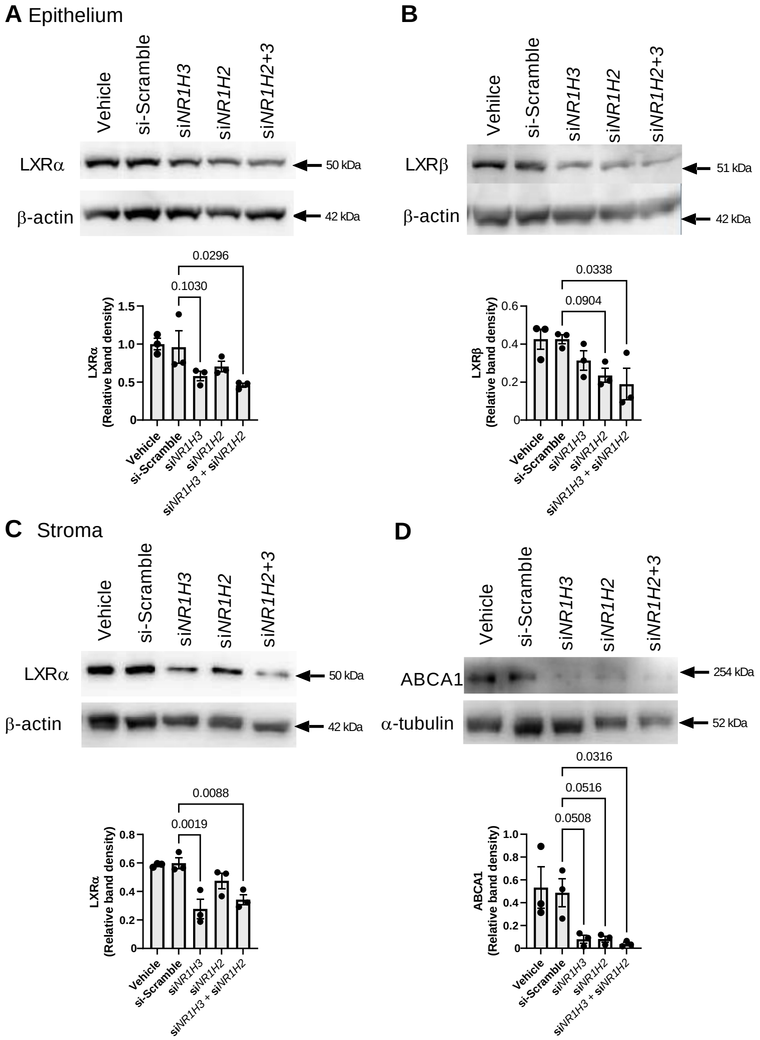

Supplemental Figure 5. siRNA targeting *NR1H3* and *NR1H2* reduces the abundance of LXRα, LXRβ and ABCA1 protein.

Epithelial (A, B) and stromal (C, D) cells were transfected for 48 hours with scramble siRNA or siRNA targeting *NR1H3*, *NR1H2*, or both *NR1H3* and *NR1H2*, and analyzed by Western blot for LXRα and LXRβ (epithelial cells), or LXRα and ABCA1 (stromal cells; Western blots for LXRβ in stromal cells had staining that precluded analysis), with β-actin or α-tubulin as loading controls. Densitometry data (lower panels) for LXRα and LXRβ are normalized to β-actin, and densitometry data for ABCA1 are normalized to α-tubulin. Data are presented as mean (SEM) using cells from 3 independent animals; statistical significance was determined using one-way ANOVA and P values reported for Dunnett’s post hoc test.
