# Supplementary material for: Oxysterols protect bovine endometrial cells against pore‐forming toxins from pathogenic bacteria
Source: FASEB J. 2021 Sep 27;35(10):e21889. doi: 10.1096/fj.202100036R (PMC9272411; doi:10.1096/fj.202100036R)
Supplement: Supplementary file 6 — Fig S6 [file FSB2-35-e21889-s006.pdf]

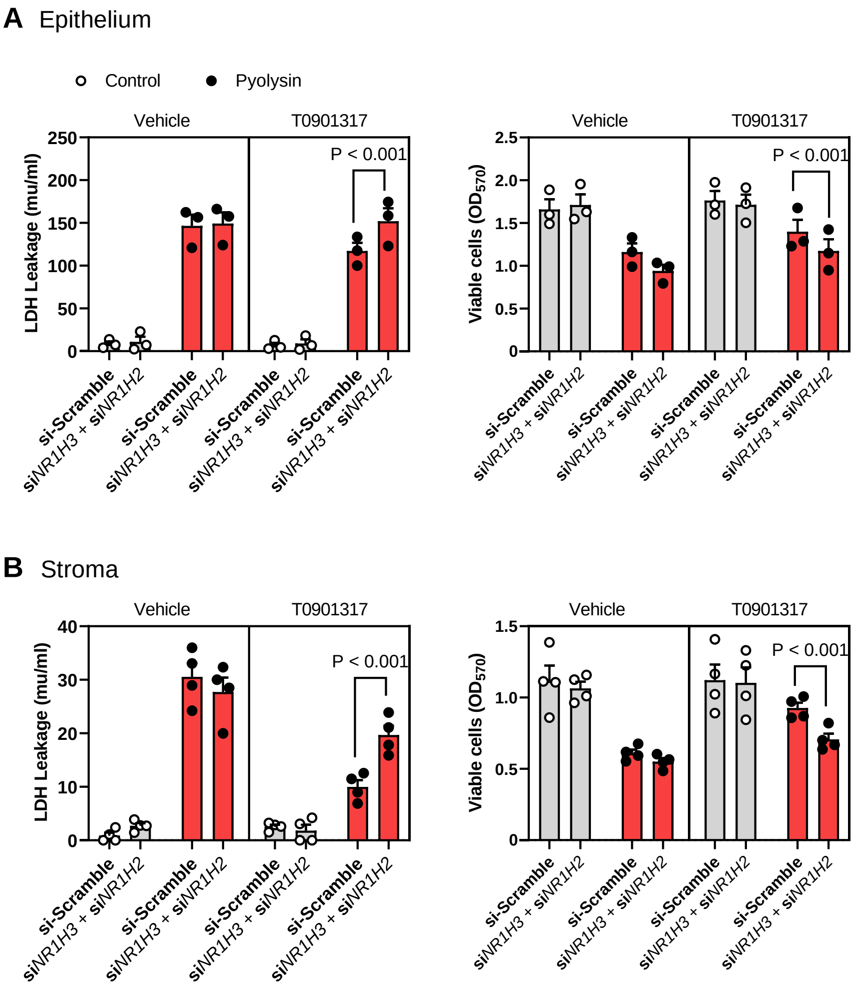

Supplemental Figure 6. T0901317 cytoprotection depends on *NR1H3* and *NR1H2*.

Epithelial (A) and stromal (B) cells were transfected for 48 hours with scramble siRNA or siRNA targeting both *NR1H3* and *NR1H2*; cultured for 24 hours in serum-free medium containing vehicle or 25 nM T0901317; and, then challenged for 2 hours with control medium (■) or pyolysin (■, epithelium 200 HU, stroma 25 HU). The leakage of LDH into cell supernatants was measured, and cell viability was determined by MTT assay. Data are presented as mean (SEM) using cells from ≥ 3 independent animals; statistical significance was determined using two-way ANOVA and Tukey's post hoc test.
